# Spatiotemporal variation in population dynamics of a narrow endemic, *Ranunculus austro‐oreganus*

**DOI:** 10.1002/ajb2.16446

**Published:** 2024-12-17

**Authors:** Riley D. Thoen, Lauren B. Hendricks, Graham T. Bailes, Bart R. Johnson, Laurel Pfeifer‐Meister, Paul B. Reed, Bitty A. Roy, Megan L. DeMarche

**Affiliations:** ^1^ Department of Plant Biology University of Georgia Athens GA USA; ^2^ Environmental Studies Program University of Oregon Eugene OR USA; ^3^ Jefferson County Open Spaces Golden CO USA; ^4^ Institute of Ecology and Evolution, Department of Biology University of Oregon Eugene OR USA; ^5^ Department of Landscape Architecture University of Oregon Eugene OR USA; ^6^ Institute for Applied Ecology Corvallis OR USA

**Keywords:** demography, integral projection model, life table response experiment, narrow endemic, Ranunculaceae, skewed growth, structured population model, vital rates

## Abstract

**Premise:**

Understanding how population dynamics vary in space and time is critical for understanding the basic life history and conservation needs of a species, especially for narrow endemic species whose populations are often in similar environments and therefore at increased risk of extinction under climate change. Here, we investigated the spatial and temporal variation in population dynamics of *Ranunculus austro‐oreganus*, a perennial buttercup endemic to fragmented prairie habitat in one county in southern Oregon.

**Methods:**

We performed demographic surveys of three populations of *R. austro‐oreganus* over 4 years (2015–2018). We used size‐structured population models and life table response experiments to investigate vital rates driving spatiotemporal variation in population growth.

**Results:**

Overall, *R. austro‐oreganus* had positive or stable stochastic population growth rates, though individual vital rates and overall population growth varied substantially among sites and years. All populations had their greatest growth in the same year, suggesting potential synchrony associated with climate conditions. Differences in survival contributed most to spatial variation in population growth, while differences in reproduction contributed most to temporal variation in population growth.

**Conclusions:**

Populations of this extremely narrow endemic appear stable, with positive growth during our study window. These results suggest that populations of *R. austro‐oreganus* are able to persist if their habitat is not eliminated by land‐use change. Nonetheless, its narrow distribution and synchronous population dynamics suggest the need for continued monitoring, particularly with ongoing habitat loss and climate change.

Population dynamics of species vary over space and time, and identifying what drives this variation in performance is fundamental to species conservation and our understanding of population ecology (Morris and Doak, 2002; Angert, 2006; Wion et al., 2020; Conquet et al., 2023). Spatial variation in population dynamics may reveal environmental gradients affecting population growth, which can be useful for conservationists to identify favorable environmental conditions (Merinero et al., 2020; Shefferson et al., 2020; Römer et al., 2021; Buckley and Puy, 2022). On the other hand, assessing population dynamics over time can unveil important variability in performance and identify its drivers within a population (Nicolé et al., 2011; Germain and Lutz, 2020; Lindell et al., 2022). For example, high variability in population dynamics over time can reduce long‐term performance of the population (i.e., the stochastic population growth rate *λ*_s_; Morris and Doak, 2002) and influence the evolution of life history strategies of a species (Hastings and Caswell, 1979; Williams et al., 2015). Finally, the degree to which spatially separated populations experience correlated fluctuations in population dynamics over time can profoundly impact the conservation of that species as a whole (Koenig and Liebhold, 2016; Hansen et al., 2020). Identifying the vital rates (e.g., survival, growth, and reproduction) underlying spatial and temporal variation in population growth rate (*λ*) can provide insight into appropriate management strategies for populations and the basic biology of species.

The degree to which variation in population dynamics affects a species' persistence may depend in part on aspects of its distribution, such as geographic range size and occupancy. Temporal environmental variation that influences population growth can reduce the stochastic population growth rate and increase the risk of local extinction (Morris and Doak, 2002). If two populations grow in similar conditions from year to year, their population dynamics may exhibit temporal synchrony, where population growth rates fluctuate in parallel among years (Moran, 1953; reviewed by Liebhold et al., 2004). Temporal synchrony is particularly a problem for species with narrow geographic ranges (and thus are at higher risk of extinction risk than widespread species; Thomas et al., 2004; Išik, 2011; Rota et al., 2022) because geographically clustered populations are more likely to live in similar environmental conditions (Grøtan et al., 2005; Di Cecco and Gouhier, 2018). For example, Dibner et al. (2019) showed that the degree of temporal synchrony among subpopulations of the rare endemic *Yermo xanthocephalus* decreased sharply with spatial distance along an elevational gradient. Additionally, if species with small ranges also have low occupancy (e.g., few populations per unit area; Rabinowitz, 1981), there is a greater proportional loss if a single population goes extinct. Thus, characterizing the temporal variation in *λ* within populations is critical to determining local extinction risk, and identifying whether this variation is synchronous among populations is an important step in scaling population dynamics to species‐level dynamics for narrow‐ranged species.

Population dynamics are often assessed using structured population models (Caswell, 2001; Morris and Doak, 2002; Doak et al., 2021). Structured population models combine vital rates into estimates of population growth (*λ*), which allows practitioners to assess the trajectory of populations. Because estimates of *λ* are derived from underlying vital rates, structured population models can also illuminate the life history and biology of species, giving them an advantage over count‐based approaches. For instance, perturbation analyses of structured population models can identify which vital rates have the largest proportional and absolute influence on *λ* (elasticity and sensitivity, respectively), which can guide conservation practitioners in allocating resources (i.e., prospective analyses; Caswell, 2000; Griffith, 2017). Models can also be decomposed to test which vital rates contributed to observed variation in *λ* across years or sites by multiplying a vital rate's sensitivity by the difference between the mean vital rate and its value in a given site or year (i.e., a life table response experiment, or retrospective analysis; Caswell, 2000; Griffith, 2017). Since structured population models are so flexible, a wide variety of vital rate model structures can be integrated into them. For instance, in nature, the growth distribution of a population may be skewed due to shrinkage or herbivory, though skewed growth is rarely included in structured population models (DeMarche et al., 2019). If skewness of growth is modeled directly, prospective and retrospective analyses can be used to determine how much that specific metric might impact population growth. Lastly, structured population models can estimate the lifespan of a species, which can be critical for determining the timeline of the species' extinction risk (Doak et al., 2021). Hence, structured population models are a powerful tool for understanding the biology of a rare species and assessing conservation risk.

In this study, we assessed the population dynamics of three populations of *Ranunculus austro‐oreganus* L.D. Benson (Ranunculaceae) across 4 years to understand how vital rates and *λ* vary in space and time for this narrow endemic. *Ranunculus austro‐oreganus* is an herbaceous perennial native to prairie habitat and confined to a single county in southern Oregon (Cruzan et al., 2021; Appendix S1: Figure S1). Possible threats to this species include climate change, herbivory, habitat loss, and introgression from a widespread congener (*Ranunculus occidentalis*; Cruzan et al., 2021). We asked: Are these natural populations in decline? How do different vital rates contribute to variation in *λ* across populations and years? And do the populations of this narrow endemic experience synchronous fluctuations in vital rates and λ among years, indicating common environmental drivers?

## MATERIALS AND METHODS

### Study system

*Ranunculus austro‐oreganus* is a narrow‐endemic, perennial herb growing in fragmented prairie and oak savanna in Jackson County, Oregon, USA, which sits in a mediterranean climate zone with cool, wet winters and hot, dry summers. It occupies a range of approximately 1400 km^2^, but there are many populations of *R. austro‐oreganus* within the range (69 occurrences separated by ≥500 m), and populations are locally abundant (NatureServe Explorer, 2024). It likely originated recently via peripatric speciation from its widespread congener *R. occidentalis* and has maintained its narrow range since the speciation event (Cruzan et al., 2021). *Ranunculus austro‐oreganus* produces showy, yellow‐orange flowers that are likely bee‐ and fly‐pollinated, similar to other *Ranunculus* species, though their flowers are also capable of self‐fertilization (Cruzan et al., 2021). Seeds germinate in the fall and grow throughout the winter as vegetative rosettes. Seedlings generally do not flower in their first year in the field, but they can do so in a greenhouse (Cruzan et al., 2021; Reed et al., 2021). Reproductive plants flower in late February to late April and set seed mid‐April to early May (Reed et al., 2019, 2021). Plants become dormant for the dry summer months before re‐emerging during the fall. *Ranunculus austro‐oreganus* may possess a persistent seed bank because previous field studies have found low germination rates (Reed et al., 2021) and other *Ranunculus* spp. do exhibit seed dormancy (Sarukhán, 1974). We found little evidence for adult dormancy during spring months (see Results).

We surveyed *R. austro‐oreganus* at three sites in Jackson County: Denman Prairie (Denman), Upper Table Rock (UpperTable), and Roxy Ann Peak (Roxy; Appendix S1: Figure S1; Appendix S2: Table S1). Denman and UpperTable are less than 1 km from one another and are situated near the western edge of the range; Roxy is ~15 km from either site but is located centrally in the *R. austro‐oreganus* range (Appendix S1: Figure S1). The sites differ in a number of environmental characteristics (Hendricks, 2016). Roxy (949 m a.s.l.) is at a higher elevation than Denman (378 m a.s.l.) and UpperTable (418 m a.s.l.), and Roxy is historically cooler and wetter than Denman and UpperTable (Appendix S1: Figure S2; Appendix S2: Table S1). All sites are located on slight slopes, in open prairie and oak savanna. Roxy has much deeper soils than Denman and UpperTable (>80 cm vs. 29 cm and 17 cm, respectively; Appendix S2: Table S1). Soils at UpperTable have higher carbon and nitrogen content and are made up mostly of sand and silt; Roxy soil has low soil carbon and nitrogen and higher clay (Appendix S2: Table S1). Carbon, nitrogen, and clay content in Denman soils are intermediate of UpperTable and Roxy (Appendix S2: Table S1). *Ranunculus occidentalis* is also widespread at Denman and UpperTable and has hybridized with *R. austro‐oreganus*, but introgressed alleles from *R. occidentalis* are present at all sites (Cruzan et al., 2021).

### Data collection

In 2015, we established 1 or 2 permanent, 1‐m‐wide transects within each site and marked approximately 200 individuals of *R. austro‐oreganus* (Appendix S2: Table S1). We also marked five large individuals outside the transect to ensure we captured the full range of size variation at each site. We measured vital rates on each marked individual for 4 years (2015–2018) to parameterize a size‐based integral projection model (IPM; Merow et al., 2014). Each year during fruit set, we scored survival and counted the total number of leaves per plant and measured the length of the longest leaf blade (Appendix S1: Figure S3). We used the natural log of the product of leaf number and leaf length as our measure of size (as done by Reed et al., 2021). We also scored whether a plant was reproductive (i.e., it produced ≥1 flower) and counted the total number of flowers produced by a plant. Flowers or entire inflorescences were sometimes lost to grazing; we only counted flowers that persisted to the end of the year. However, to explore patterns of herbivory, we also counted the total number of stems missing per plant (e.g., if there was a broken or missing stem). Based on personal observations (B. A. Roy), missing stems were largely due to herbivory by white‐tailed deer.

To obtain estimates of recruitment, we performed seedling censuses each spring from 2016 to 2018. We placed 625‐cm^2^ quadrats at regular intervals along each transect (mean = 40 quadrats/site/year, range = 23–65). We counted newly emerged plants in each quadrat and scored them as seedlings if they were within a known seedling size distribution and did not flower (distribution obtained from data in Reed et al., 2021). *Ranunculus austro‐oreganus* may produce new clonal rosettes via belowground rhizomes, but these are generally distinguishable from seedlings based on size (B. R. Johnson, personal observations). New plants outside the seedling size distribution and/or that flowered in the first year were considered adults that were clones or were previously missed, and their vital rates were included with other adult vital rates. Only 12% of new plants were classified as adults. We scored survival of newly emerged seedlings to their second year and measured the size of surviving recruits in their second year.

To supplement our demographic data, we downloaded historic and contemporary climate data from the PRISM grid cells containing our study sites (PRISM Climate Group, 2023). We downloaded a 118‐year time series of minimum, maximum, and mean monthly temperature and total precipitation between January and July of each year, as these were the earliest and latest months with Global Biodiversity Information Facility (GBIF) records of *R. austro‐oreganus* (GBIF.org, 2023). We visualized these data to aid in interpretation of population and year differences in population growth (Appendix S1: Figure S2). Additionally, we plotted the number of missing stems per plant to assess herbivore damage among sites and years (Appendix S1: Figure S4).

### Data analyses

All data analysis were conducted in R version 4.0.2 (R Core Team, 2020).

#### Vital rate models

For transitions of adult plants, we fit size‐dependent vital rate models for growth, survival, probability of flowering, and flower number. We modeled growth from size in time *t* to *t* + 1 as a generalized additive model (GAM; GAMLSS version 5.3‐4; Rigby and Stasinopoulos, 2005) with a skewed normal distribution to account for asymmetric growth (e.g., shrinkage; DeMarche et al., 2019). Here, the mean (*µ*), standard deviation (*σ*), and skewness (*ν*) of the size distribution in time *t* + 1 was modeled jointly with potentially distinct sets of independent variables for each parameter. We used generalized linear models (GLMs) with binomial error distributions to model survival from year *t* to *t* + 1 and incidence of flowering in year *t*. We modeled flower number with a negative binomial distribution to account for overdispersion. In our study, less than 4% (42 of 1064) of marked plants were recorded as not present during a survey only to be found the next year, suggesting rare adult dormancy, premature rosette death or herbivore removal, or sampling error. We did not model adult dormancy due to limited data, and we did not monitor seed dormancy.

For adult vital rate models (growth mean, variance, and skewness, survival, probability of flowering, and flower number), we fit a maximal model with a three‐way interaction of size in time *t*, site, and year as explanatory variables, and used the corrected Akaike information criterion (AICc) to select the best nested model (Burnham and Anderson, 2002) using the dredge() function in the MuMIn package (version 1.43.17; Bartón, 2020). To predict vital rate responses accurately while avoiding overfitting models, we selected the simplest model within 2 ΔAICc of the minimum (Tredennick et al., 2021).

We included seedlings as an additional stage class in our IPM and thus modeled seedling vital rates independent of size. To calculate the rate of seedling production per flower (the flower‐seedling transition), we divided seedling density at each site by the overall flower density at that site in the previous year. We modeled the number of seedlings produced per flower as a linear model and seedling survival as a GLM with binomial error. There was no evidence of skewness in seedling size in their second year, so we modeled the size of surviving recruits as a GAM with a normal distribution to allow joint estimation of the mean (*µ*) and standard deviation (*σ*).

We observed a limited number of seedlings (see Results) to estimate seedling survival and growth (mean and variance), so we fit maximal models for these vital rates with just site as a predictor due to limited sampling across years. For seedling production per flower, we included both site and year as main effects. We selected the best model within two ΔAICc of the minimum model using the dredge() function.

#### Integral projection model

We built IPMs to combine vital rate models to project population growth rates at each site for three annual transitions (three sites × three transitions = nine IPMs). The IPMs were constructed according to a pre‐reproductive census (Rees et al., 2014), wherein the transition rates of each size class represent the sum of a survival/growth kernel and a reproduction kernel characterized by the product of the flowering probability, number of flowers per reproductive plant, and predicted number of seedlings per flower (Eqs. 1 and 2).

(1) $$n(y,t+1)=\int_{L}^{U}[S\left(x\right)G\left(y,x\right)+G_{S}\left(y\right)S_{s}B\left(t\right)]n(x,t)\mathrm{dx}$$

(2) $$B\left(t+1\right)=\int_{L}^{U}\left[P_{f}\left(x\right)f_{w}\left(x\right)P_{r}\right]n\left(x,t\right)\mathrm{dx},$$

where *S*(*x*) is the probability of survival of a plant with starting size *x*, *G*(*y*,*x*) is the growth of an adult plant of starting size *x* to size *y*, *G*_s_(*y*) is the size distribution of 1‐year‐old plants, *S*_s_ is the survival of seedlings to become 1‐year‐old plants, and *B*(*t*) is the number of seedlings in year *t*, *P*_f_(*x*) is the probability of a plant of size *x* flowering, *f*_w_(*x*) is the number of flowers produced by a plant of size *x* conditional on flowering, and *P*_r_ is the number of seedlings that recruit per flower; *L* is the lower and *U* the upper limit of the natural log of the product of leaf number and longest leaf length for the whole data set.

We discretized kernels into 100 evenly divided size classes and included a separate seedling stage class. We used the median of each size class to discretize vital rate functions, which has been shown to increase accuracy of *λ* estimates (Doak et al., 2021). However, we used midpoints to discretize the five largest size classes, which had few representatives from natural populations. We estimated growth probabilities of adults and surviving seedlings using the difference between the cumulative density function (CDF) of upper and lower bounds of size classes (Doak et al., 2021). We avoided growth eviction (i.e. ., losing individuals because their growth is outside of the defined range) by re‐normalizing the probability density function of growth to sum to 1.0 (Williams et al., 2012).

We calculated the deterministic population growth rate (*λ*) for each site and annual transition as the dominant eigenvalue of each discretized projection matrix (Rees et al., 2014). We also calculated stochastic population growth rates (*λ*_s_) for each site through stochastic simulation using the stoch.growth.rate() function in the popbio package with 5000 iterations (popbio version 2.7; Stubben and Milligan, 2007). We estimated the lifespan of *R. austro‐oreganus* by simulating a starting population of 100 seedlings using the mean survival/growth kernel (i.e., ignoring reproduction) and observing the timespan until 99% of individuals had died.

#### Perturbation analyses

We performed perturbation analyses on vital rate model coefficients to understand how individual vital rates contributed to population growth rate. To calculate sensitivity, elasticity, and life table response experiment (LTRE) contributions, we created mean vital rate models by averaging site and year regression coefficients. Then, we used these mean vital rate models to generate a mean projection kernel. To perturb vital rate model coefficients, we added 0.00001 to the intercept and size slope of each mean model and calculated sensitivity and elasticity values for each vital rate (Griffith, 2017).

We performed a life table response experiment to retrospectively explore which vital rates drove observed differences in *λ* among sites and years. We decomposed the variation in *λ* among sites and years into LTRE contributions of each vital rate model coefficient (e.g., adult survival intercept and size slope). We calculated LTRE contributions as the product of the difference in a vital rate model coefficient for a particular site and year relative to the mean value and the sensitivity of *λ* to that coefficient (Griffith, 2017). Because LTRE values are additive, we present LTRE contributions for each vital rate model (e.g., summing contributions from intercepts and slopes). In the main text, we focus on variation across sites and years separately.

#### Estimating error

To obtain estimates of error around all outputs, we performed a parametric bootstrap incorporating both model and parameter uncertainty. We randomly sampled candidate vital rate models from a 95% confidence set with probability equal to the relative AICc weight. We then resampled coefficients from the selected model from a multivariate normal distribution with the model's estimated variance–covariance matrix. We repeated this process for 5000 iterations to construct bias‐corrected 95% confidence intervals around estimates of *λ*, *λ*_s_, elasticity, sensitivity, and LTRE contributions. Among years within each site, we took the pairwise difference in *λ* over each iteration and took pairwise differences in *λ*_s_ among sites. When the null hypothesis was not within the bounds of our bootstrapped confidence intervals (e.g., 1 for *λ*, and 0 for perturbation analyses and pairwise differences in *λ* or *λ*_s_), we considered the responses to have a statistically significant effect.

## RESULTS

### Vital rate models

Size was included as a predictor in all of the most parsimonious adult vital rate models, as were main effects of site and year (Appendix S1: Figures S5–S8; Appendix S2: Tables S2–S5; Appendix S3). Inclusion of interactions in the best model differed across vital rates. Site × year interactions were included in growth mean, growth variance, survival, flowering probability, and flower count models, indicating that temporal variation differed among sites. Size × year was in growth mean, growth variance, growth skew, survival, and flowering probability, and size × site interactions were included in growth mean, growth variance, and flowering probability. Size × year and size × site interactions indicated temporal or spatial variation depended on plant size, respectively. The most parsimonious seedling vital rate models only included an intercept (e.g., no site or year effects; Appendix S1: Figures S9–S11; Appendix S2: Tables S2, S6–S8).

### Population growth rates

**Figure 1:**
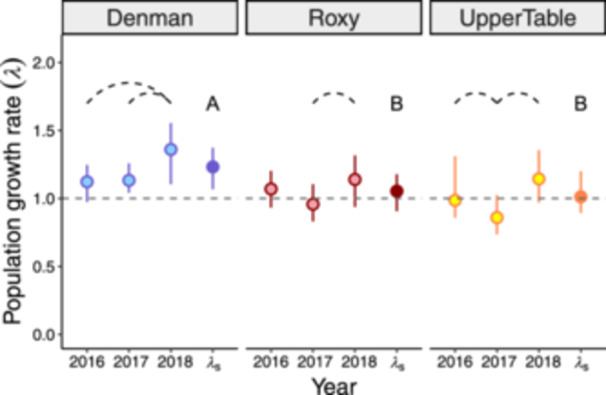
Yearly deterministic population growth rates (*λ*) of *Ranunculus austro‐oreganus* across three sites over 3 years, and the stochastic population growth rate *λ*_s_ for each site. Error bars represent bias‐corrected 95% CIs. The year represents the second year of a transition. Dashed curves indicate significant differences in *λ* among years within a site; different letters indicate significant differences in *λ*_s_ among sites. The dashed horizontal line at 1 indicates a stable population.

Asymptotic population growth rates varied both spatially and temporally but were always either positive or not significantly different from 1.0, indicating population growth or stability (Figure 1). In all three years, *λ* at Denman was above 1.0, and significantly so in 2017 and 2018 (*λ*_2017_ = 1.13, 95% CI: [1.04, 1.26]; *λ*_2018_ = 1.36, 95% CI: [1.11, 1.55]); overall, *λ*_s_ at Denman was above 1.0, suggesting long‐term population growth (*λ*_s_ = 1.23, 95% CI: [1.07, 1.37]; Figure 1). Although both Roxy and UpperTable had individual years of population growth and decline, neither *λ* nor *λ*_s_ values were significantly different from 1.0 (Roxy *λ*_s_ = 1.05, 95% CI: [0.91, 1.18]; UpperTable *λ*_s_ = 1.01, 95% CI: [0.89, 1.20]; Figure 1), suggesting long‐term population stability at these sites. Stochastic population growth rate at Denman was significantly greater than that of Roxy and UpperTable (Figure 1).

All three sites demonstrated similar changes in population growth rates across years, though statistically significant differences varied among sites. All sites had their highest *λ* in 2018, and both Roxy and UpperTable exhibited their lowest *λ* in 2017 while Denman had nearly identical *λ* in 2016 and 2017 (Figure 1). At all three sites, *λ* was significantly greater in 2018 than in 2017 (Figure 1). Both Denman and UpperTable had additional differences in *λ* among years, with 2018 higher than 2016 at Denman, and 2016 higher than 2017 at UpperTable (Figure 1). We estimated the maximal lifespan for a new seedling as 22 years (95% CI: 18, 24).

Roxy was cooler and wetter than the other two sites over our study period (Appendix S1: Figure S2). Additionally, 2016–2017 was the wettest for all sites over our study period, while 2017–2018 was the driest. For all sites, 2016–2017 was the coolest transition period, whereas 2015–2016 was the warmest. Finally, fewer than 15% of stems were missing during each site‐year, with 2016 at Denman and UpperTable having the greatest rates of stems missing (Appendix S1: Figure S4).

### Perturbation analyses

**Figure 2:**
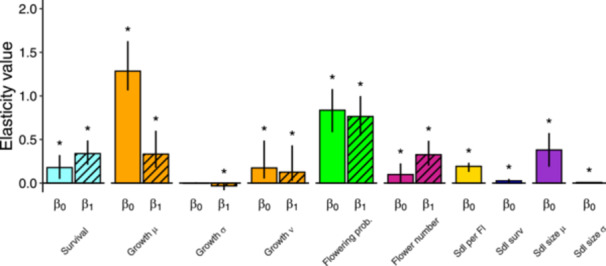
Elasticity values (±95% bias‐corrected CIs) of vital rate model coefficients for *Ranunculus austro‐oreganus*. Filled bars denote the intercept for each vital rate; hatched bars denote the size slope of adult vital rate models. Elasticities are the proportional change in *λ* of the mean integral projection model given a perturbation to mean model coefficients; a higher elasticity indicates greater proportional influence on λ. CIs were calculated by parametric bootstrapping. Asterisks denote when 95% CI does not overlap 0. For abbreviated vital rates in the figure, prob. = probability, sdl = seedling, and surv = survival. *β*_0_ = vital rate model intercepts, *β*_1_ = vital rate model slopes, *μ* = mean size estimate, *σ* = size variance, *ν* = growth skewness.

Prospective analyses identified growth and reproduction as the vital rates with the strongest effect on *λ*. Population growth was most elastic to adult plant growth and flowering probability (Figure 2). The growth model intercept had the highest elasticity, followed by flowering probability intercept and flowering probability size slope. Seedling growth, survival slope, growth slope, and flower number slope also had large proportional effects on *λ* (Figure 2). The variance in adult growth had a small negative elasticity; all other vital rate coefficients had positive effects on *λ*. Sensitivity analysis also indicated strong effects of adult plant growth and reproduction, as well as adult survival (Appendix S1: Figure S12). Growth slope and flower count slope had the largest positive sensitivities, followed by flowering probability slope and survival slope. The growth variance slope had a considerable negative sensitivity. Interestingly, sensitivities of model slopes were higher than intercepts across the board, which we interpret as larger plants having a greater absolute contribution to *λ* via each vital rate (Appendix S1: Figure S12). All elasticity and sensitivity estimates were significantly different from 0, indicating that each vital rate had consistently positive or negative effects across bootstraps (Figure 2; Appendix S1: Figure S12).

**Figure 3:**
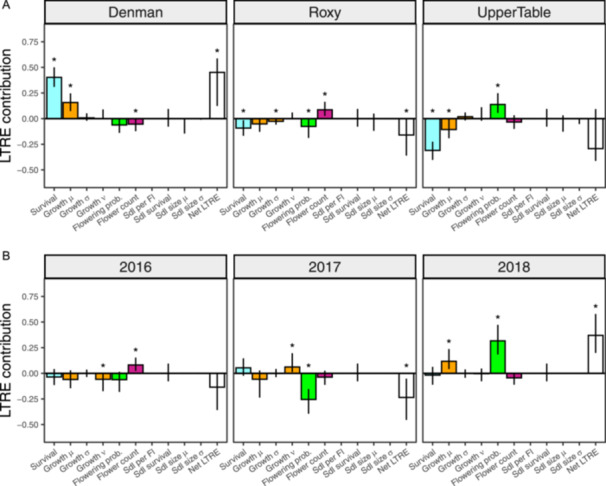
Life table response experiment contributions (LTRE) (±95% bias‐corrected CIs) of vital rates across (A) sites and (B) years. LTRE values describe the contribution of different vital rates to variation in *λ* across sites and years; a positive contribution means that vital rate had a positive influence on *λ* in that site or year relative to the mean integral projection model. Asterisks denote when 95% CI does not overlap 0, indicating a significant contribution. LTRE contributions for each vital rate model were summed across the intercept and slope, and contributions were summed across years in A and across sites in B. See Appendix S1: Figure S13 for LTRE contributions over each site‐year. *μ* = mean size estimate, *σ* = size variance, *ν* = growth skewness.

Retrospective analyses indicated differences in *λ* between sites was primarily driven by changes in survival, growth, flowering probability, and flower count (Figure 3A). Differential survival across sites contributed the most to spatial variation in *λ*, with a significant positive LTRE contribution at Denman (0.40, 95% CI: [0.31, 0.50]) and significant negative contributions at UpperTable (–0.31, 95% CI: [–0.40, –0.25]) and Roxy (–0.09, 95% CI: [–0.17, –0.02]). Mean growth also had a significant positive LTRE contribution at Denman (*µ*: 0.16, 95% CI: [0.07, 0.25]) and negative contributions at UpperTable (*µ*: –0.10, 95% CI: [–0.19, –0.01]); growth variance had a negative contribution at Roxy (*σ*: –0.02, 95% CI: [–0.06, 0]). Reproductive vital rates (flowering probability and flower count) differed in the direction of their LTRE contributions across sites. Flowering probability contributed positively to *λ* at UpperTable but negatively at Roxy (significant) and Denman (not significant). On the other hand, flower count contributed negatively at Denman (significant) and UpperTable (not significant), but positively at Roxy (Figure 3A).

Differences in *λ* between years was primarily driven by changes in growth and reproductive vital rates (Figure 3B). Flowering probability had the strongest LTRE contributions across years, with a negative contribution in 2016 (–0.06, 95% CI: [–0.18, 0.15]) and 2017 (0.26, 95% CI: [–0.39, –0.15]), and a strong positive contribution in 2018 (0.32, 95% CI: [0.18, 0.47]). Flower count had small LTRE contributions across years, only producing a significant positive contribution in 2016 (0.08 [0.02, 0.15]). Interestingly, the mean and skewness of growth had significant LTRE contributions across years; growth skewness had a significant negative contribution in 2016 (*ν*: –0.06 [–0.17, –0.01]) and positive contribution in 2017 (*ν*: 0.06 [0.01, 0.20]. Growth mean had a positive significant LTRE contribution in 2018 (*µ*: 0.12 [0.04, 0.24]) but had negative contributions in 2016 and 2017, though these were not significant (Figure 3B). Despite being the strongest driver of differentiation in *λ* across sites, survival had little impact on differences in *λ* across years. There was limited evidence for novel site × year interactions in LTRE contributions (i.e., LTRE values summed across site and year represent most of the variation in all site × year combinations; Appendix S1: Figure S13).

## DISCUSSION

All three populations of *R. austro‐oreganus* were either stable or growing across our study period. However, we did uncover spatial and temporal variation in adult vital rates that contributed to variable population growth among years and sites. Interestingly, the dominant vital rates driving spatial variation differed from those driving temporal variation. Survival was the primary driver of differences in *λ* among sites, while flowering probability was the foremost driver of variation in *λ* among years. We also found some evidence of temporal synchrony in *λ* among sites, suggesting that common environmental drivers may explain variation in *λ* from year to year. Below, we discuss spatial and temporal variation in population growth and vital rates, and their consequences for conservation of *R. austro‐oreganus*.

### Spatial variation of population growth

We found differences in stochastic population growth rate (*λ*_s_) among sites over our study period; while the population at Denman was growing, those at Roxy and UpperTable appeared to be stable (Figure 1). Site was included as a main effect in all the best adult vital rate models, suggesting that all vital rates differed among sites on average (Appendix S1: Figures S5–S8; Appendix S2: Tables S2–S5; Appendix S3). However, our LTRE analysis suggests that adult survival played a primary role in explaining site differences in *λ* despite a relatively weak effect in prospective analyses (Figure 2; Appendix S1: Figure S12). Higher survival, and to a lesser extent faster growth, contributed to the higher population growth observed at Denman, while decreases in these vital rates contributed to the lower population growth at Roxy and UpperTable. Reproductive vital rates showed a compensatory pattern, with higher flowering probability at UpperTable and greater flower production at Roxy partially compensating for the lower survival and growth at these sites.

These differences in vital rates and *λ*_s_ among sites could be driven by any number of environmental and evolutionary differences among sites. Interestingly, Denman and UpperTable differed in population dynamics despite their proximity, indicating geographic distance alone cannot explain differences in population dynamics. Rather, differential physical characteristics of sites that impact plants at small spatial scales may have generated the differences between these two nearby sites. For example, Denman is a lower‐elevation site with deeper and clayey soils (Appendix S2: Table S1). Indeed, Nicolé et al. (2011) found differential population growth among nearby sites (some <10 km) of the endangered *Dracocephalum austriacum*. In this study, different topographic slopes among nearby sites affected the survival of *D. austriacum* and generated differences in *λ*, pointing to the effects of microenvironmental variation on *λ* even despite proximity (Nicolé et al., 2011). Proximity does not warrant equivalence of population dynamics, and these studies, along with our own, highlight the need to monitor many sites to assess the spatial variance in a species' population dynamics.

### Temporal synchrony in population growth

We found *λ* varied among years at each site in our study, and that for each adult vital rate, year was included in the best model (Appendix S1: Figures S5–S8; Appendix S2: Tables S2–S5; Appendix S3). We observed some synchrony in *λ* among sites; in most years, the three sites had similar shifts in *λ*. For example, all three sites had significantly higher *λ* in 2018 than in 2017 (Figure 1). These corresponding shifts in population growth suggest some common environmental factor(s) impacted the three sites similarly within a year, which is perhaps unsurprising given the small geographic range of *R. austro‐oreganus* (Liebhold et al., 2004; Koenig and Liebhold, 2016). If a common driver is responsible for the synchronous swings in *λ* among the three sites, then an increase in that driver's variability (reducing *λ*_s_) or shifts toward values that reduce *λ* could lead to correlated declines among populations. Although any number of factors could be driving temporally synchronous fluctuations in *λ*, we discuss below the potential for climate and herbivory to be important drivers in this system.

Each site experienced similar temperature and precipitation fluctuations in the *R. austro‐oreganus* growing season relative to its 100+ year average conditions at that site (Appendix S1: Figure S2), and these fluctuations corresponded to synchrony in *λ*. Specifically, the driest and coolest year corresponded to the highest *λ* at all sites (2018), whereas the wettest year was the year with the lowest overall *λ* (Figures 1, 3B; Appendix S1: Figure S2). Our observed relationships between weather and population dynamics correspond closely with a recent climate manipulation experiment using this species (Reed et al., 2021). Reed et al. (2021) observed significantly higher population growth of *R. austro‐oreganus* in plots with an experimental drought treatment and when experimental populations were transplanted north of the current range. However, experimental drought had a much larger effect on *λ* than transplanting north, and experimental warming increased *λ* at all latitudes, suggesting precipitation is a stronger driver of *λ* in *R. austro‐oreganus* than is temperature (Reed et al., 2021). One limitation of our study is that we could not account for differing seedling vital rates among years due to low sample sizes (Figure 3B). However, Reed et al. (2021) found that high seedling growth and survival mainly drove high *λ* under drought conditions, suggesting our models may have been more conservative towards capturing variation in *λ* among years.

Another potential driver of *λ* in our study was herbivory by deer. We found that the number of missing stems at each site was somewhat synchronous among years (Appendix S1: Figure S4). The number of missing stems corresponded little with variation in *λ* (Figure 1 and Appendix S1: Figure S3), but the LTRE analysis identified changes in the growth skewness that correspond with changes in missing stems (Figure 3; Appendix S1: Figure S4; discussed below). In a recent meta‐analysis, Morris et al. (2020) found that biotic drivers such as herbivory may impact *λ* as much as or more than climate does. For example, *Trillium grandiflorum* population growth decreases in populations and years with high deer herbivory due to negative impacts on both growth and reproduction (Knight, 2004; Knight et al., 2009). Because the range of *R. austro‐oreganus* is so small, populations may experience similar fluctuations in the intensity of deer herbivory over time. Large herbivore populations may exhibit temporal synchrony in number and consumption of herbs in response to climate (Grøtan et al., 2005) or oak masting (Harlow et al., 1975; Wentworth et al., 1992). Therefore, monitoring the long‐term impact of deer herbivory on population growth of *R. austro‐oreganus* and how herbivory fluctuates among years may inform the long‐term management of this species.

### Importance of skewed growth in structured population models

In IPMs, growth is often modeled as normally distributed with a mean and variance. Although the mean is almost always modeled as dependent on size and potentially other explanatory variables (such as site, year, or other covariates), the variance in growth is frequently treated as a constant (e.g., by taking the residual variance from the mean growth model). Other methods do model the variance in growth either separately or simultaneously to account for size or other effects on this parameter. Only rarely, however, do IPMs deviate from the assumption of normally distributed growth to account for potential skewness in growth (but see DeMarche et al., 2019). In this study, we found strong support for skewness in adult growth, indicating that growth probabilities were not symmetrical around the mean rate. Although growth variance and skewness had minimal impact on *λ* in prospective analyses (sensitivities and elasticities; Figure 2; Appendix S1: Figure S12), they did contribute significantly to variation in *λ* among sites (variance) and years (skewness), suggesting these vital rates are important to model if one wants to accurately capture the growth dynamics of a populations. DeMarche et al. (2019) showed that modeling growth with a normal distribution (vs. a skewed normal distribution) can over‐estimate *λ* for species that undergo events causing shrinkage in individual growth. In our study, skewed growth had positive and negative LTRE contributions in 2016 and 2017, respectively. Interestingly, skewness contributions correspond to yearly variation in the number of missing stems (Figure 3B; Appendix S1: Figure S4); 2016 had the highest rate of stems removed per plant. The 2015–2016 transition saw a negative LTRE contribution of growth skewness likely due to herbivory, whereas 2016–2017 had a positive LTRE contribution for skewness in growth, likely because plants grew back to a “normal” size with limited herbivory in 2017. Here, by directly modeling growth skewness as a vital rate, we can further clarify herbivory as a potential driver of *λ* in *R. austro‐oreganus* and better capture temporal variance in *λ*.

### Conservation implications

*Ranunculus austro‐oreganus* has been classified as G3 (vulnerable; NatureServe Explorer, 2024) due to its tiny geographic range and ongoing threats of habitat loss, climate change, and introgression with *R. occidentalis*. However, to our knowledge, no previous demographic studies have evaluated the population dynamics and viability of wild populations. We found that all three study populations were either growing or stable during the 3 years of annual transitions, which suggests that this species is not in dire need of immediate intervention to stabilize or reverse in situ declines. However, we caution that this stability may not be generalizable into the future for several reasons. First, our 4‐year study, while typical of the length of many demographic studies (Crone et al., 2011), is still quite short to assess stochastic population growth. Second, we only sampled three *R. austro‐oreganus* populations, which is a fraction of the 69 distinct geographic populations on NatureServe Explorer (2024). Third, we did not test for a seed bank in this study or distinctly model clonal reproduction. Although we have limited evidence of a seed bank for *R. austro‐oreganus* and found a low rate of clonal reproduction, failing to account for these transitions can cause a miscalculation of population growth (Nguyen et al., 2019; Sanczuk et al., 2022). However, because *R. austro‐oreganus* is a perennial, *λ* was close to 1, and only 12% of newly observed rosettes fell outside of the seedling size range, we expect omitting a seed bank would have little impact besides slightly lowering *λ* at Denman (Nguyen et al., 2019; Sanczuk et al., 2022). Fourth, key environmental drivers in this system such as precipitation and deer herbivory are likely continuing to change outside of the range in our study. Crone et al. (2013) found structured population models did a poor job predicting future population growth rates due to environmental fluctuations outside the observed environment during the study period. With climate change pushing contemporary climates outside of the historical norms, we cannot realistically conclude that population dynamics of *R. austro‐oreganus* will remain on the stable trajectory we observed. However, forecasted drying of this region could facilitate population growth for *R. austro‐oreganus* because our study and that of Reed et al. (2021) found that population growth increased in drier conditions (Mote and Salathé, 2010). Of course, the effect of temperature on *R. austro‐oreganus* is still unclear, and projected wetter winters and warmer summers may counteract any positive effects of drier summers.

Conservation practitioners may use structured population models to quantify how specific conservation actions affect population dynamics. For instance, Finkelstein et al. (2010) explored the efficacy of decreasing chick mortality from lead‐based paint ingestion for the conservation of the Laysan albatross. Additional empirical data on the effects of herbivory on vital rates in *R. austro‐oreganus* would allow us to investigate how deer removal would impact *λ* (e.g., exclosures; reviewed by Russell et al., 2001). Alternatively, structured population models could be used to determine how supplementing wild populations with greenhouse‐grown plants increases *λ*, given that these supplementations would likely increase survival, growth, and reproduction (Godefroid et al., 2011; Cruzan et al., 2021). Further, our estimated lifespan of 22 years (95% CI: 18, 24) reveals that *R. austro‐oreganus* is a moderately long‐lived perennial, which could inform the timing or frequency of management efforts, without the need to follow a cohort from germination to death. Monitoring individual vital rates and constructing structured population models thus allows for greater insight into the life history and population ecology of species (Caswell, 2000; Morris and Doak, 2002).

Finally, we note that our findings of population stability over three transitions at three sites, while encouraging, do not reduce the need to ensure that enough populations exist on sites with protected conservation status. They do, however, suggest that ensuring enough populations with varied environmental conditions are protected could be sufficient for the near term.

### Consequences of introgression

We were particularly interested to see if there was evidence of adaptive introgression contributing to population growth at Denman and UpperTable. Cruzan et al. (2021) found that *R. austro‐oreganus* is inbred and suffers from high drift load compared to its widespread congener *R. occidentalis*. Despite an apparent cytoplasmic incompatibility, gene flow between these two species is resulting in adaptive introgression at some loci from *R. occidentalis* into *R. austro‐oreganus* (Cruzan et al., 2021). Frequencies of adaptive *R. occidentalis* alleles are higher in *R. austro‐oreganus* populations at Denman and UpperTable than at Roxy, and while we see higher population growth at Denman, there was no such growth at UpperTable. Of course, our data set cannot identify whether adaptive introgression is bolstering population growth, but it is interesting to consider the consequences of adaptive introgression for this species. Whitney et al. (2015) used controlled crosses to show that introgression from *Helianthus debilis* increased fitness of *Helianthus annuus*; historic introgression between these species formed *H. annuus* subsp. *texanus* and allowed *H. annuus* to expand its range into drier climates (Whitney et al., 2010, 2015). These studies highlight the potential for adaptive introgression to improve population growth, ultimately escalating to range expansion. Ongoing research on the fitness consequences of introgressed alleles in the *Ranunculus* system will inform how secondary contact may affect population persistence, speciation, and potential expansion of *R. austro‐oreganus*.

## CONCLUSIONS

*Ranunculus austro‐oreganus* populations appear stable, though they show some degree of temporal synchrony with one another. Interestingly, the vital rates contributing to spatial and temporal variation differed, highlighting the importance of evaluating both axes of variation when assessing species' population dynamics. Populations exhibited some synchrony in performance over time, possibly due to shared climatic conditions. Subsequent population monitoring is needed to assess whether these populations will remain stable under novel climates, especially as climate becomes more autocorrelated in space (Crone et al., 2013; Di Cecco and Gouhier, 2018).

## AUTHOR CONTRIBUTIONS

Funding was secured by B.A.R., L.P.‐M., and B.R.J. The study was designed by B.A.R., L.P.‐M., B.R.J., and M.L.D. B.A.R. and B.R.J. identified and established field sites. B.A.R., L.B.H., G.T.B., B.R.J., L.P.‐M., and P.B.R. collected data. R.D.T. analyzed the data and wrote the first draft of the manuscript with input from M.L.D.; all co‐authors contributed to revisions.

## Supporting information


**Appendix S1.** Supplemental Figures S1–S13.

**Appendix S2.** Supplemental Tables S1–S8.

**Appendix S3.** Supplemental Table S9.

## Data Availability

Data for all analyses are available from Figshare at https://doi.org/10.6084/m9.figshare.27068203.v1.
