# Supplementary material for: Spatiotemporal variation in population dynamics of a narrow endemic, Ranunculus austro‐oreganus
Source: Am J Bot. 2024 Dec 17;112(1):e16446. doi: 10.1002/ajb2.16446 (PMC11744433; doi:10.1002/ajb2.16446)
Supplement: Supplementary file 1 — Appendix S1. Supplemental Figures S1–S13. [file AJB2-112-e16446-s003.docx]

| Appendix S1. Supplemental figures.  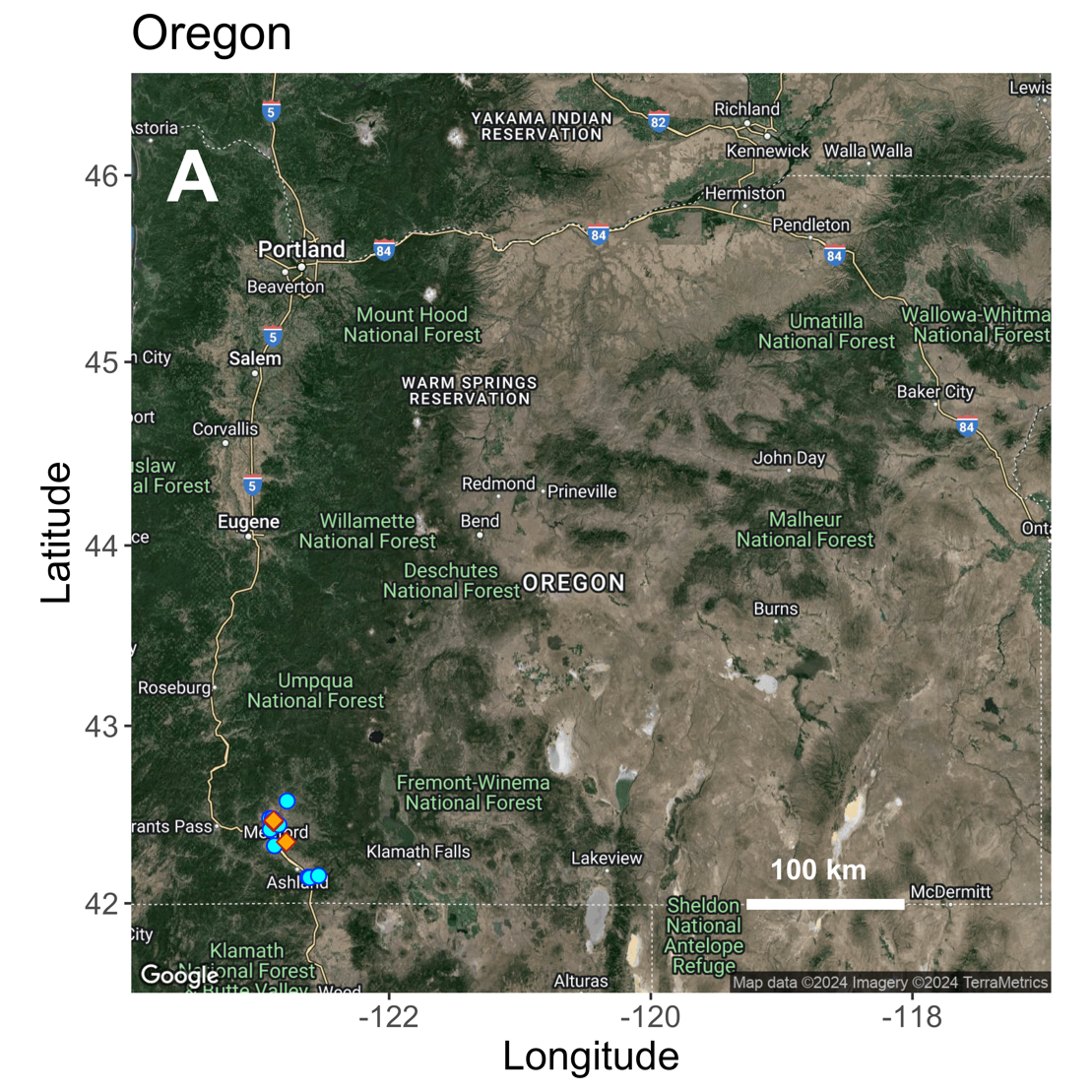 | 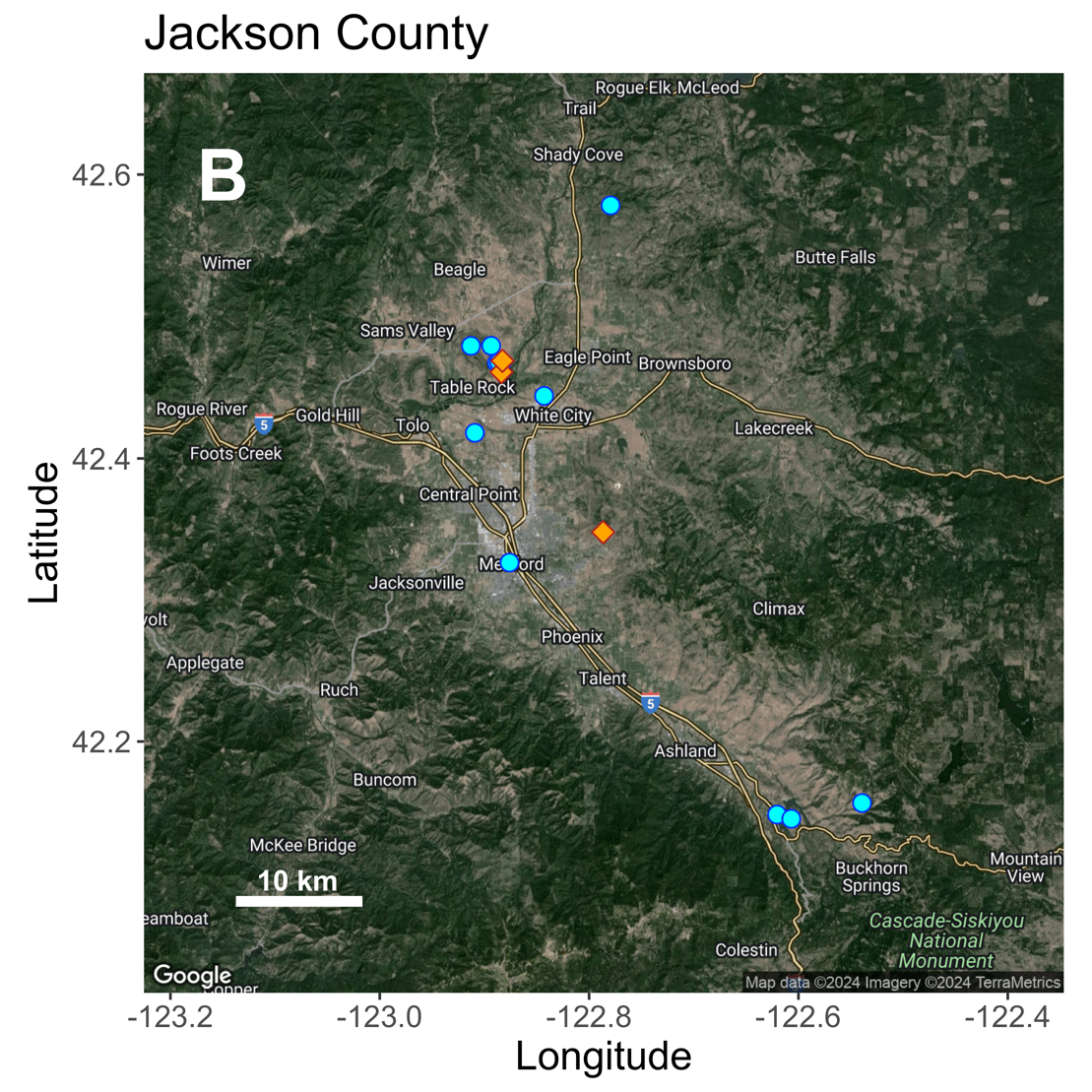 |
| --- | --- |

Figure S1. Locations of *Ranunculus austro-oreganus* GBIF records (blue) and the three sampling sites (orange). (A) Map of Oregon; the range of *R. austro-oreganus* is restricted to Jackson County, OR, near Medford, shown in (B). Plots were made with R package ggmap (Kahle and Wickham, 2013).


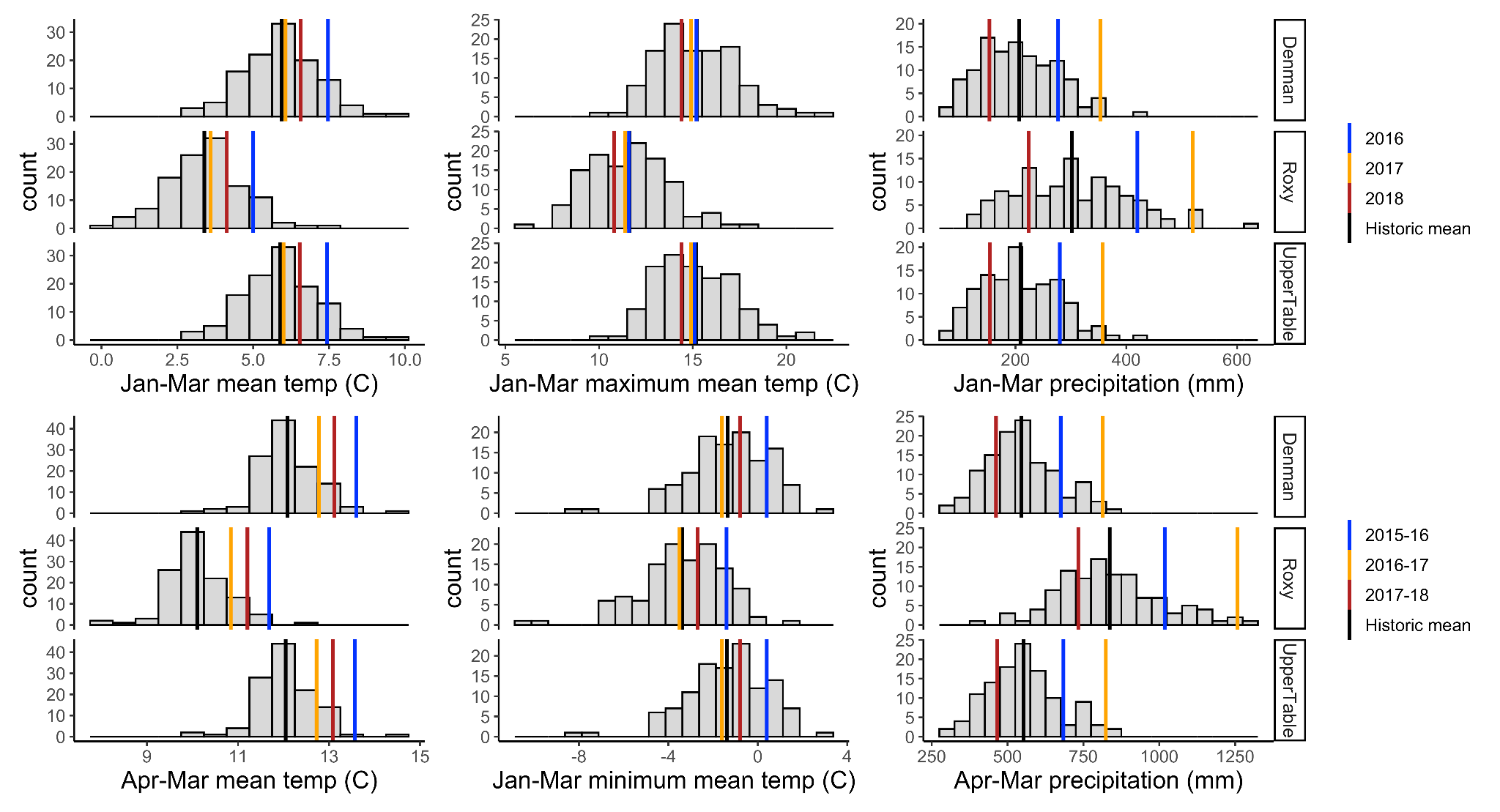


Figure S2. Historical temperature and precipitation at the three study sites. Most panes show climate variables for January to March of year *t*, but bottom left and right are from April of year *t* to March of year *t* + 1, which coincides with *R. austro-oreganus* seed set in year *t* to flowering in year *t* + 1. Historical values span 1901–2018 years and were downloaded from PRISM (PRISM Climate Group). Vertical bars show values during study years and the historical mean.


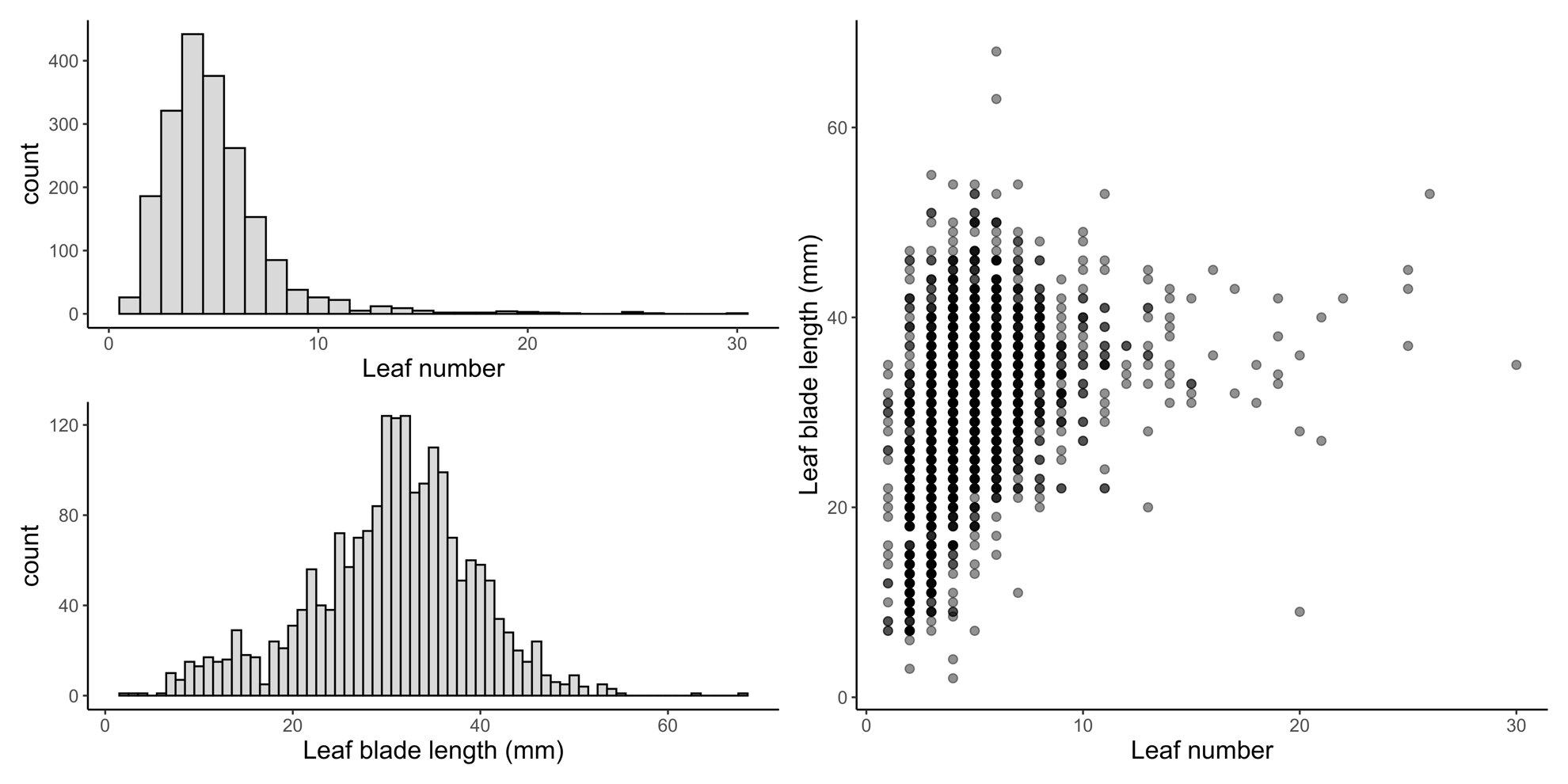


Figure S3. Variation in leaf size metrics of *Ranunculus austro-oreganus.* Top left: Histogram of leaf number, bottom left: histogram of longest leaf blade length. Right: Relationship between leaf number and longest leaf blade length. Note the final measure of size is the natural log of the product of leaf number and longest leaf blade length.


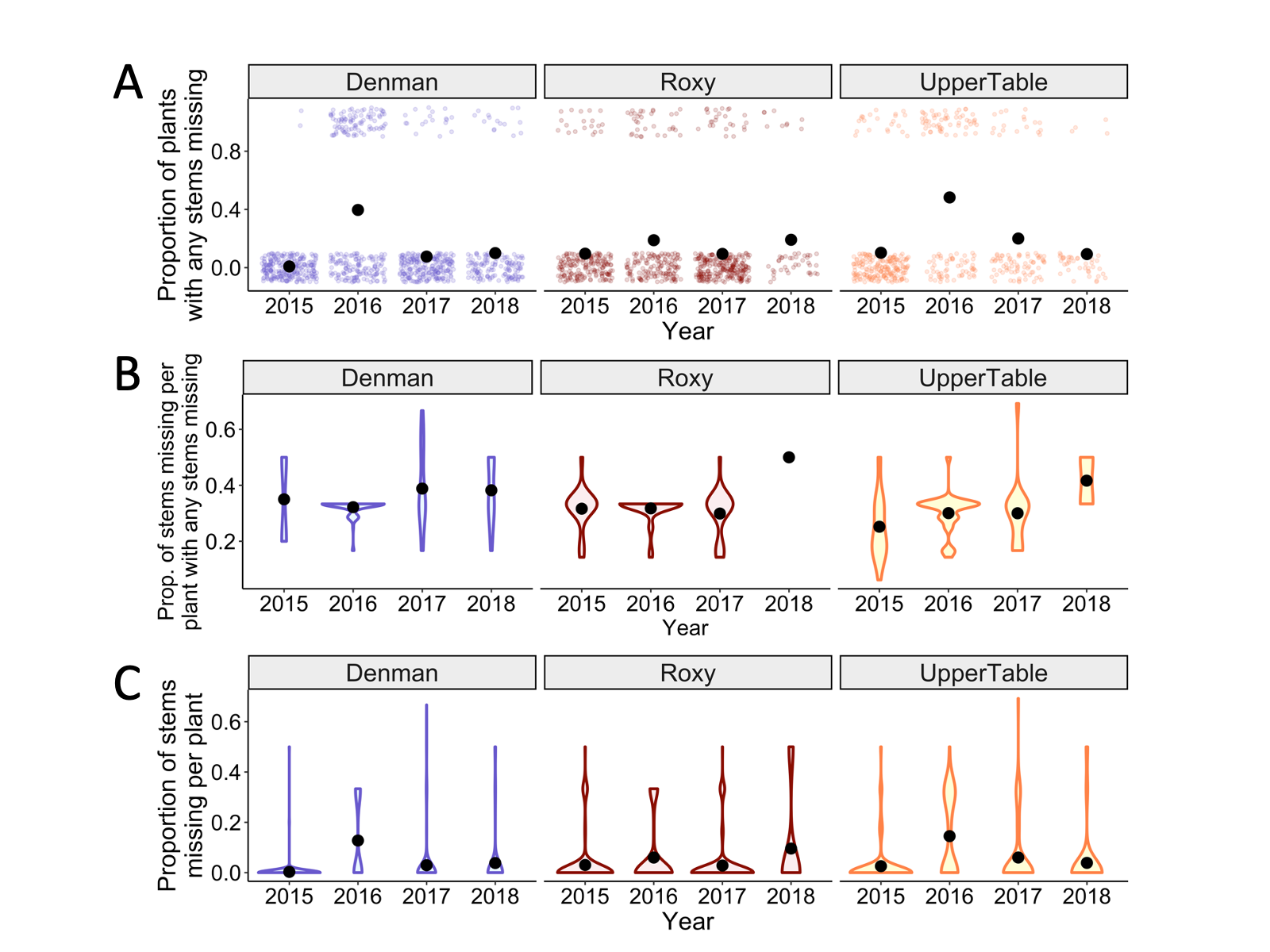


Figure S4. (A) Proportion of total flowering plants in each site and year with any stem missing (1 = has a stem gone, 0 = no stem gone). (B) Violin plot showing the proportion of stems gone per flowering plant which had any stems missing. (C) Violin plots showing the proportion of total flowering stems missing from an individual plant at each site in each year of our study (i.e. a cumulative measure of plots A and B). Black points show the mean for each site-year.


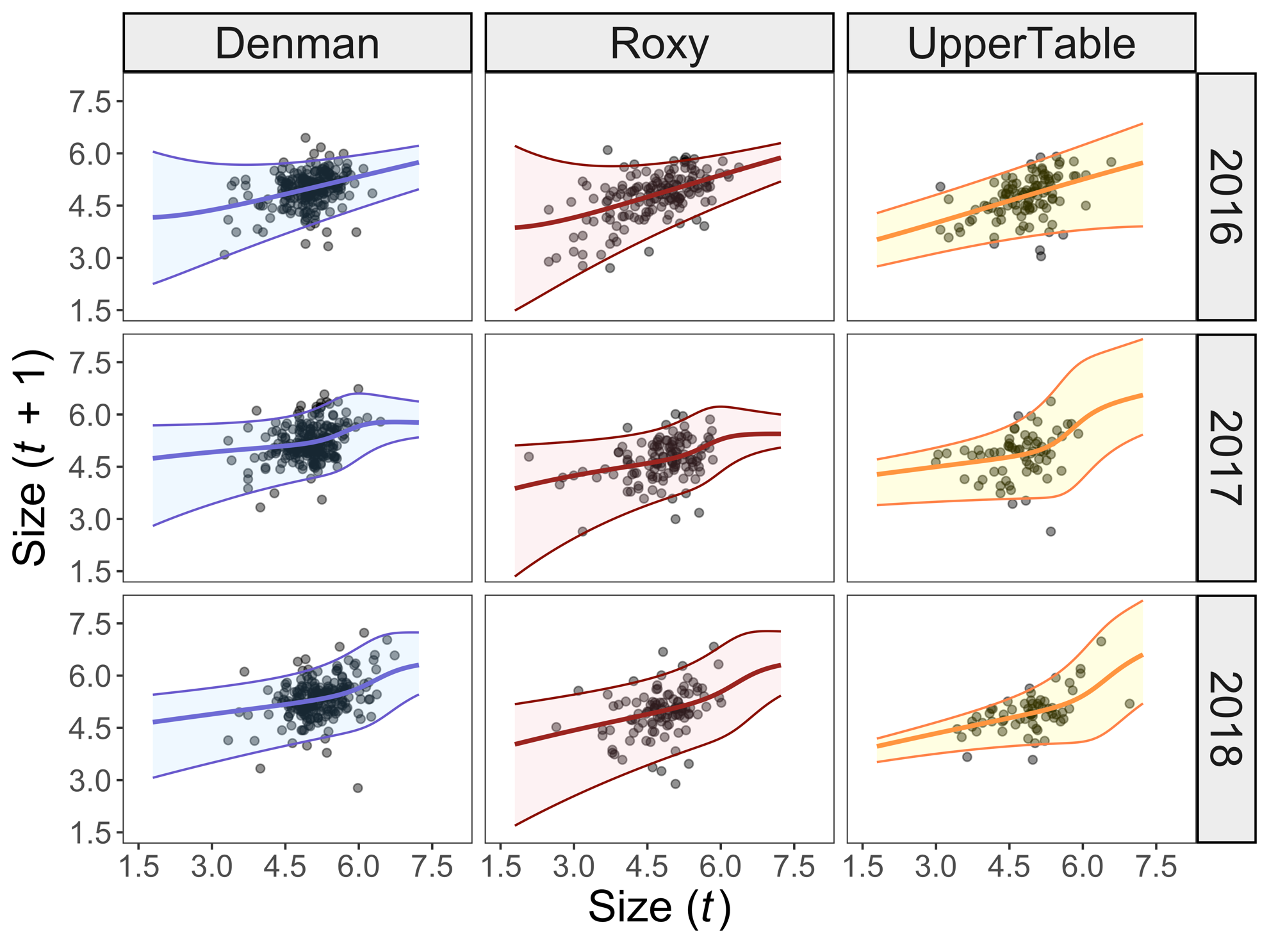


Figure S5. Growth vital rate models for each site–year combination from best-supported vital rate model. In each panel, the central line corresponds to the 50% quantile of the growth distribution; upper and lower lines are the 2.5% and 97.5% quantiles, respectively.


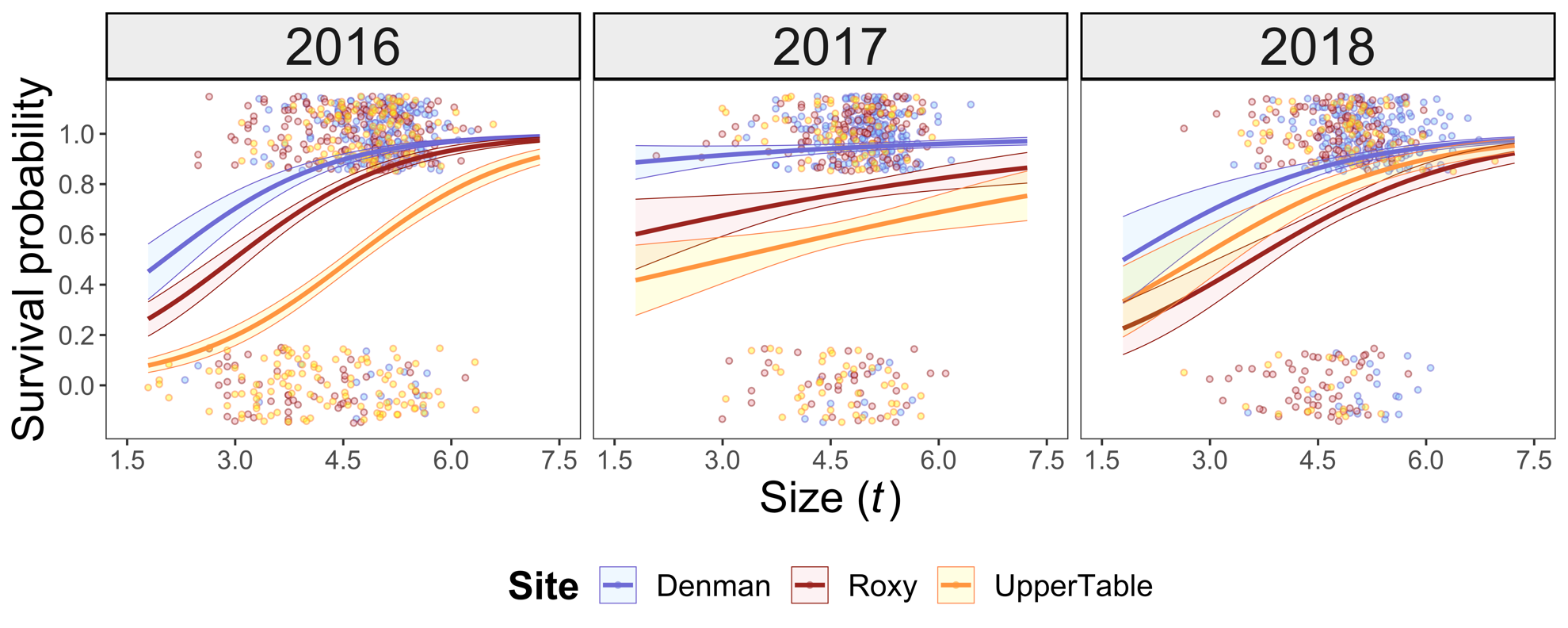


Figure S6. Survival vital rates (±SE) for each site–year combination from best-supported vital rate model. Points are jittered to prevent overlap.


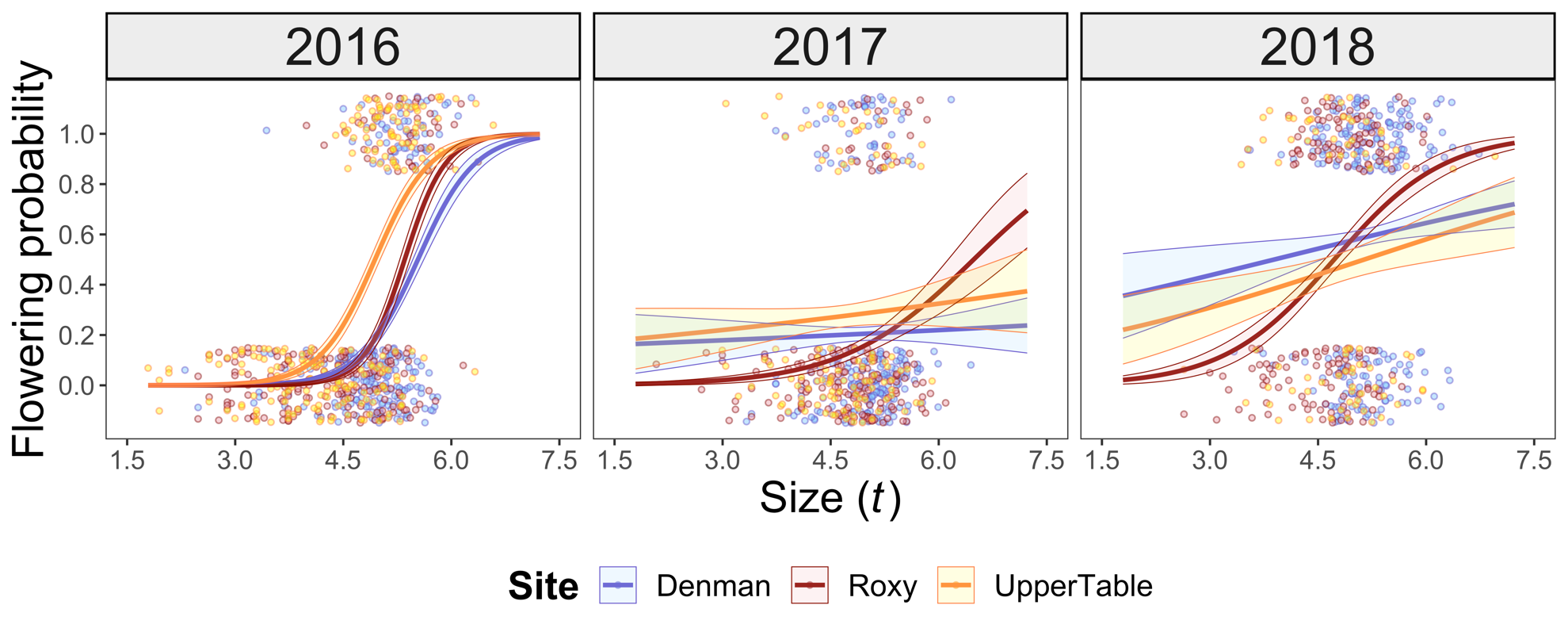


**Figure S7.** Probability of flowering (±SE) for each site-year combination from best-supported vital rate model. Points are jittered to prevent overlap.


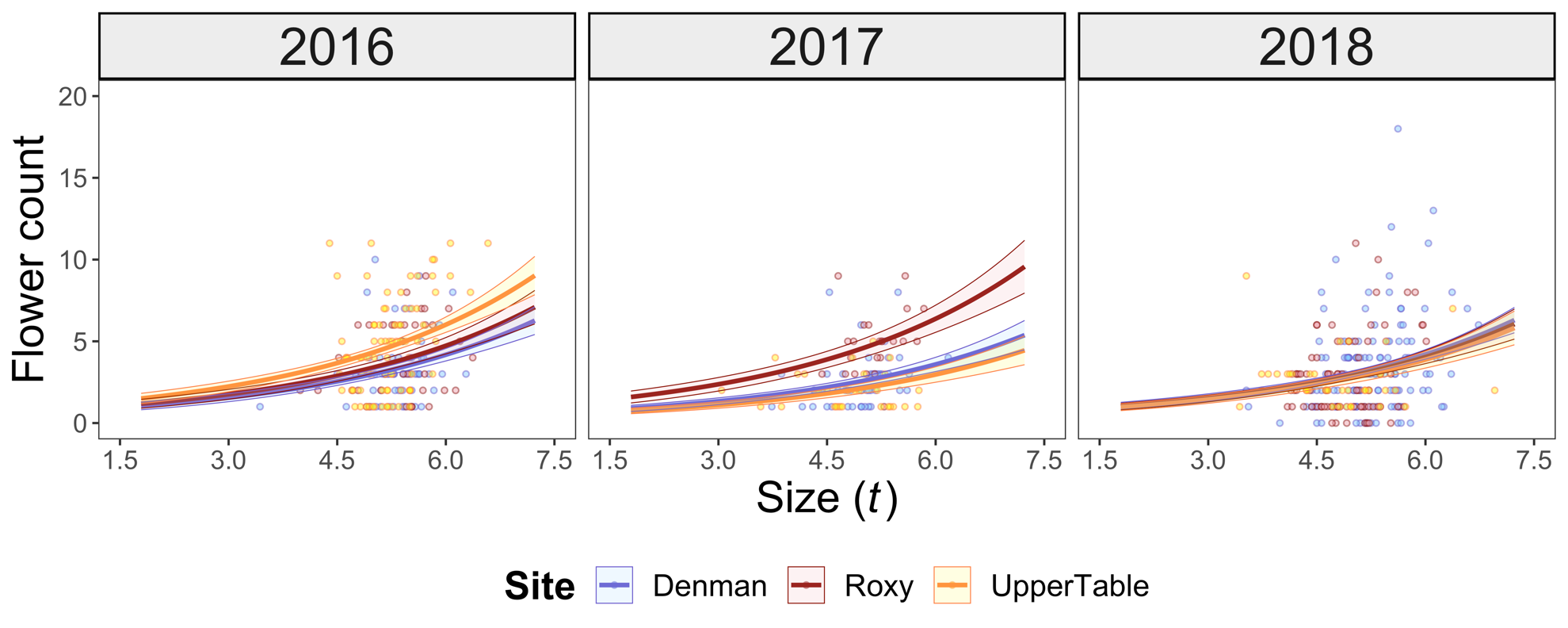


Figure S8. Flower count vital rates (±SE) for plants that flowered in each site-year combination from best-supported vital rate model.


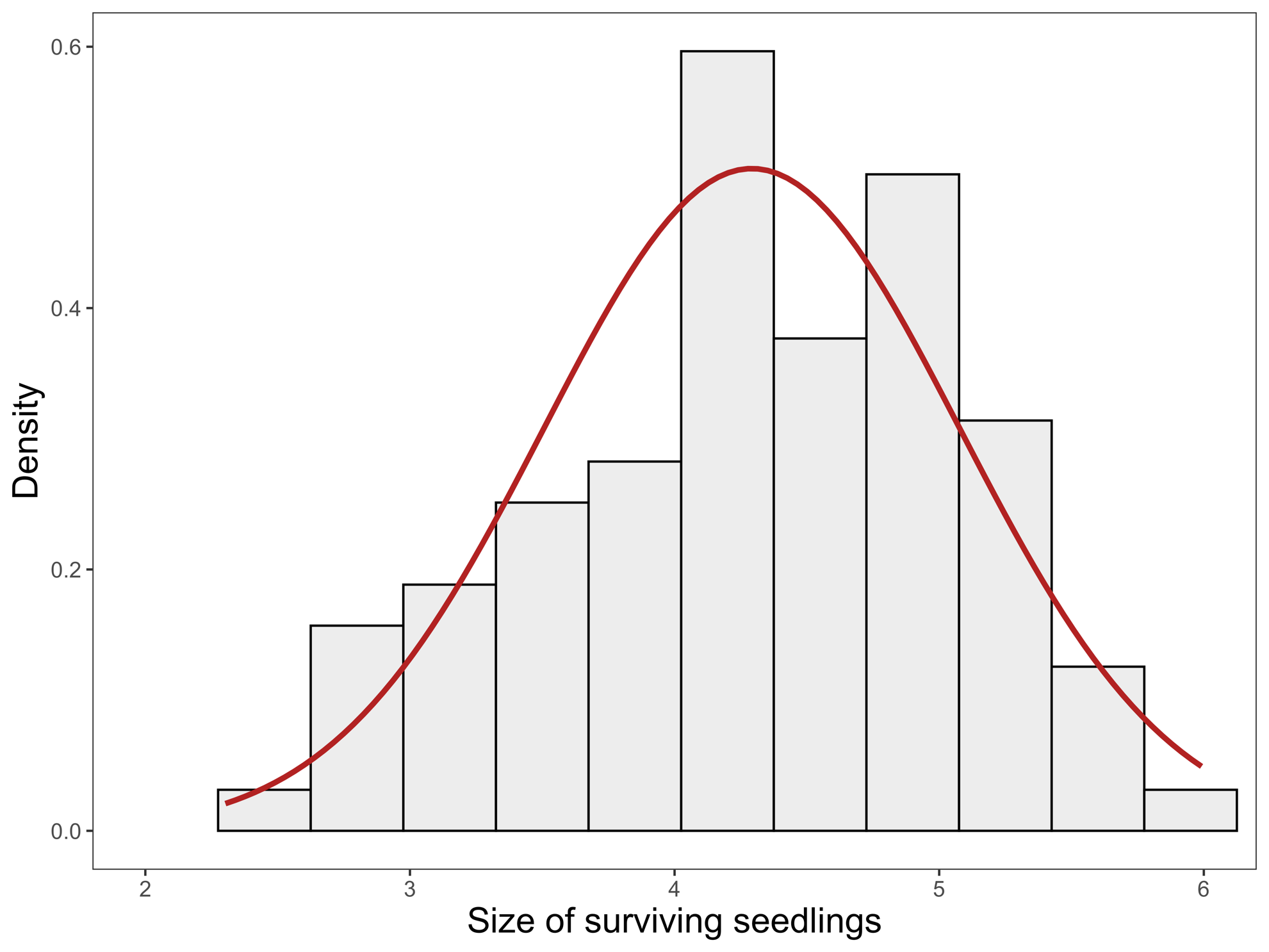


**Figure S9.** Size distribution of surviving seedlings from best-supported vital rate model. The histogram is observed seedling size; the red line is the modeled size distribution.


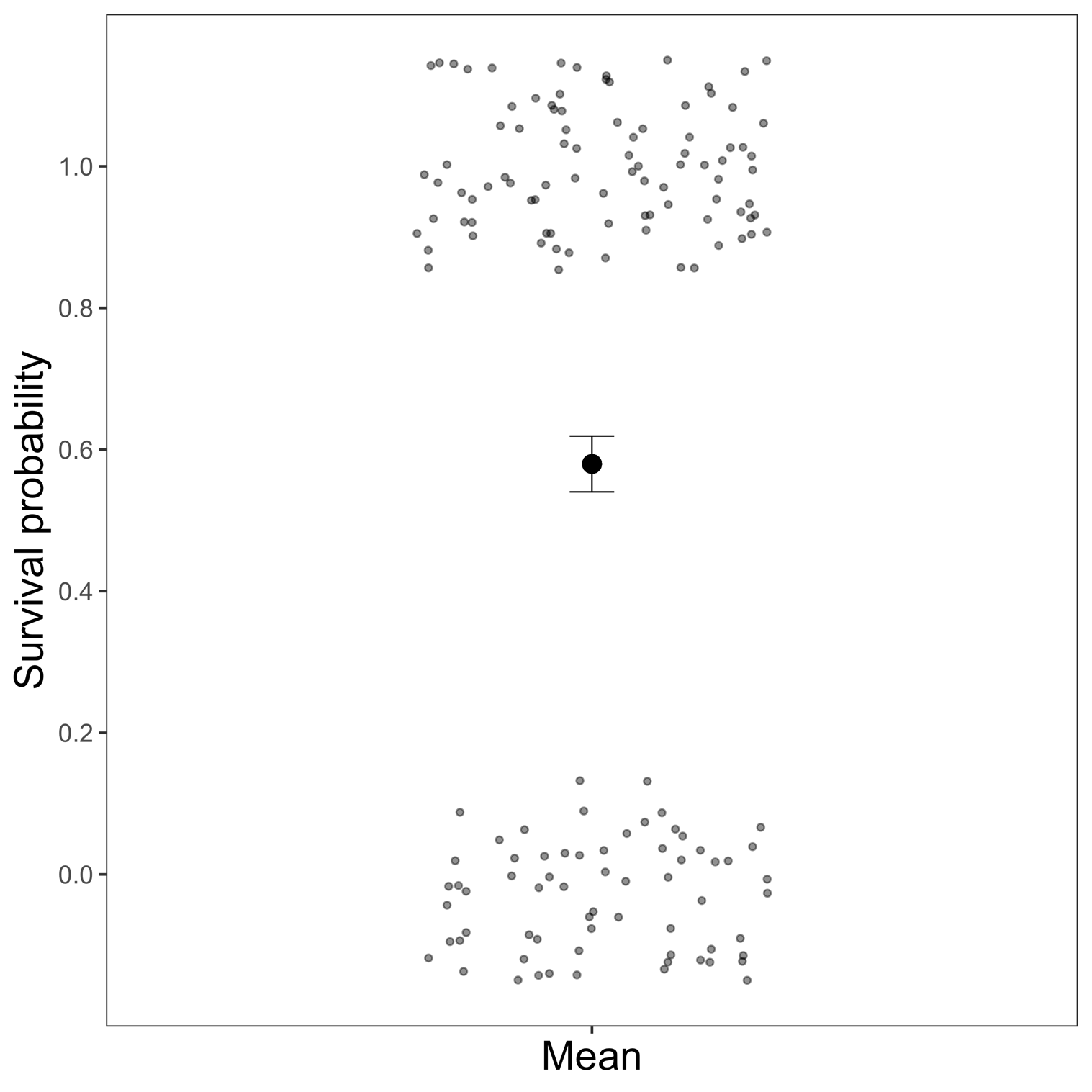


**Figure S10.** Survival (±SE) of seedlings from best-supported vital rate model. Points are jittered to prevent overlap.


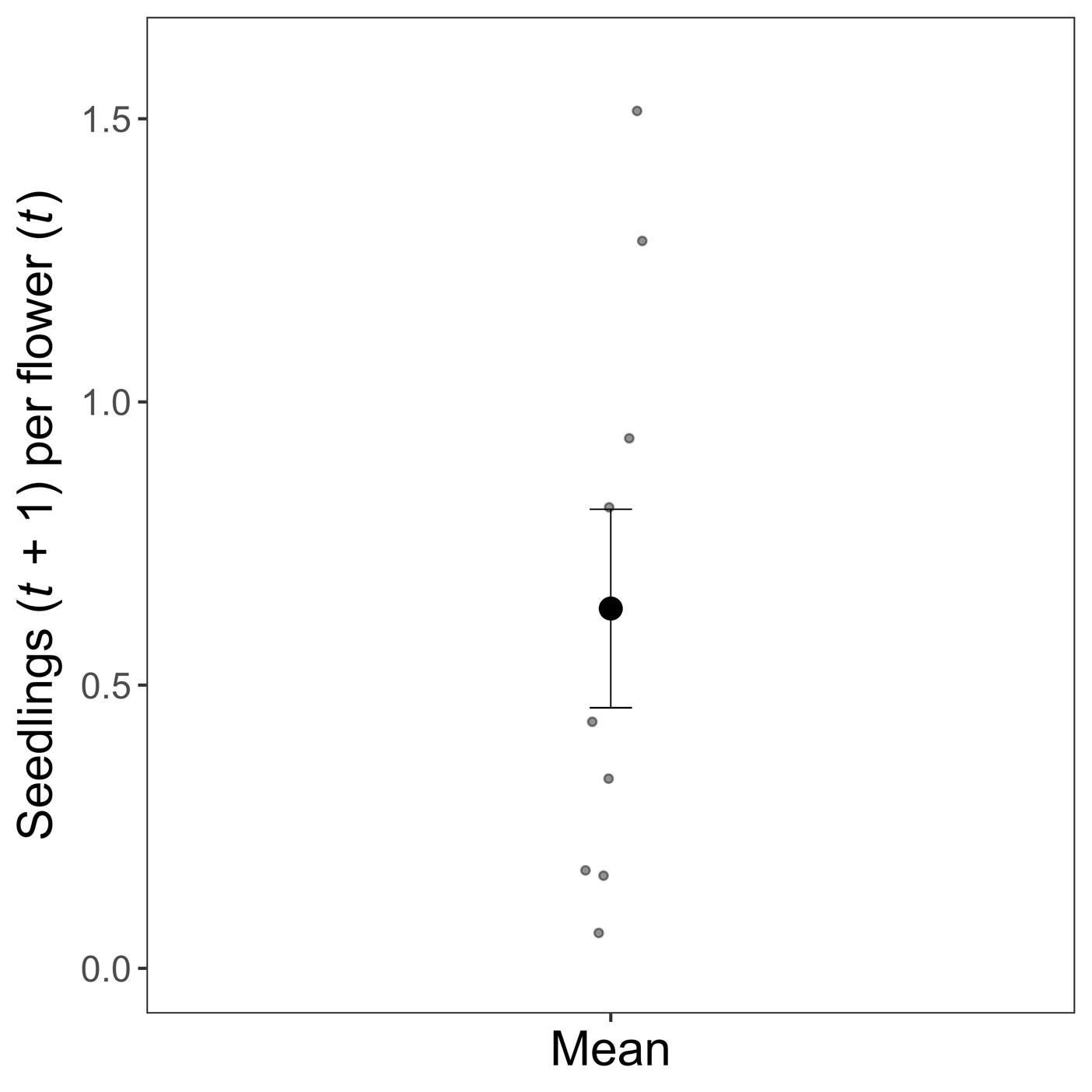


Figure S11. Number of germinants produced per flower (±SE) from best-supported vital rate model. Points are jittered to prevent overlap.


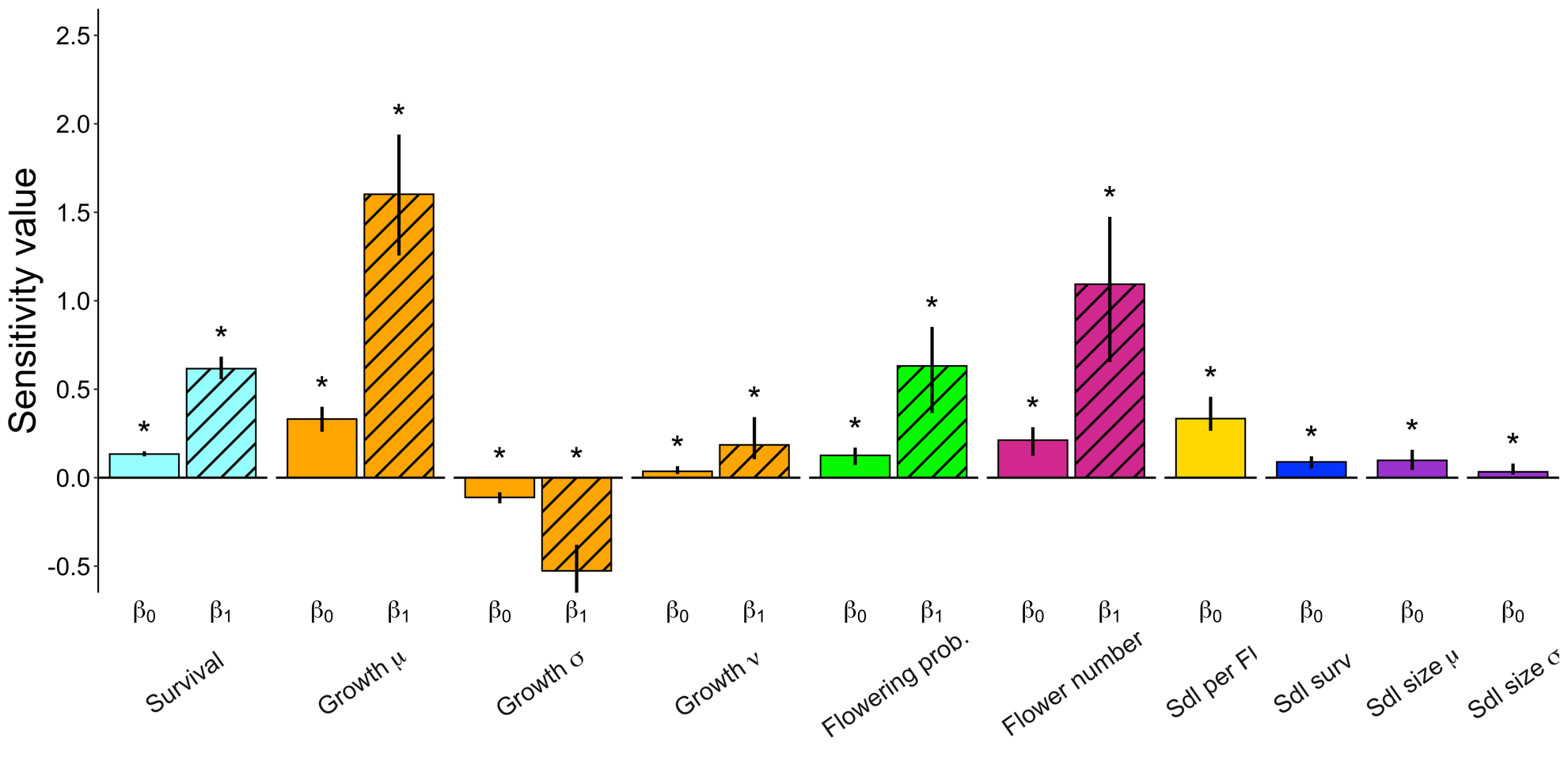


**Figure S12.** Sensitivity values (± 95% CIs) of vital rate model coefficients for *Ranunculus austro-oreganus*. Filled bars denote the intercept for each vital rate; hatched bars denote the slope size of adult vital rate models. Sensitivities are the absolute change in *λ* of the mean integral projection model given a perturbation to mean model coefficients; a higher sensitivity indicates greater influence on *λ*. Confidence intervals were calculated by parametric bootstrapping. Asterisks denote when 95% CIs do not overlap 0. For abbreviated vital rates in the figure, prob. = probability, sdl = seedling. *β*_0_ = vital rate model intercepts, *β*_1_ = vital rate model slopes, *μ* = mean size estimate, *σ* = size variance, *ν* = growth skewness.


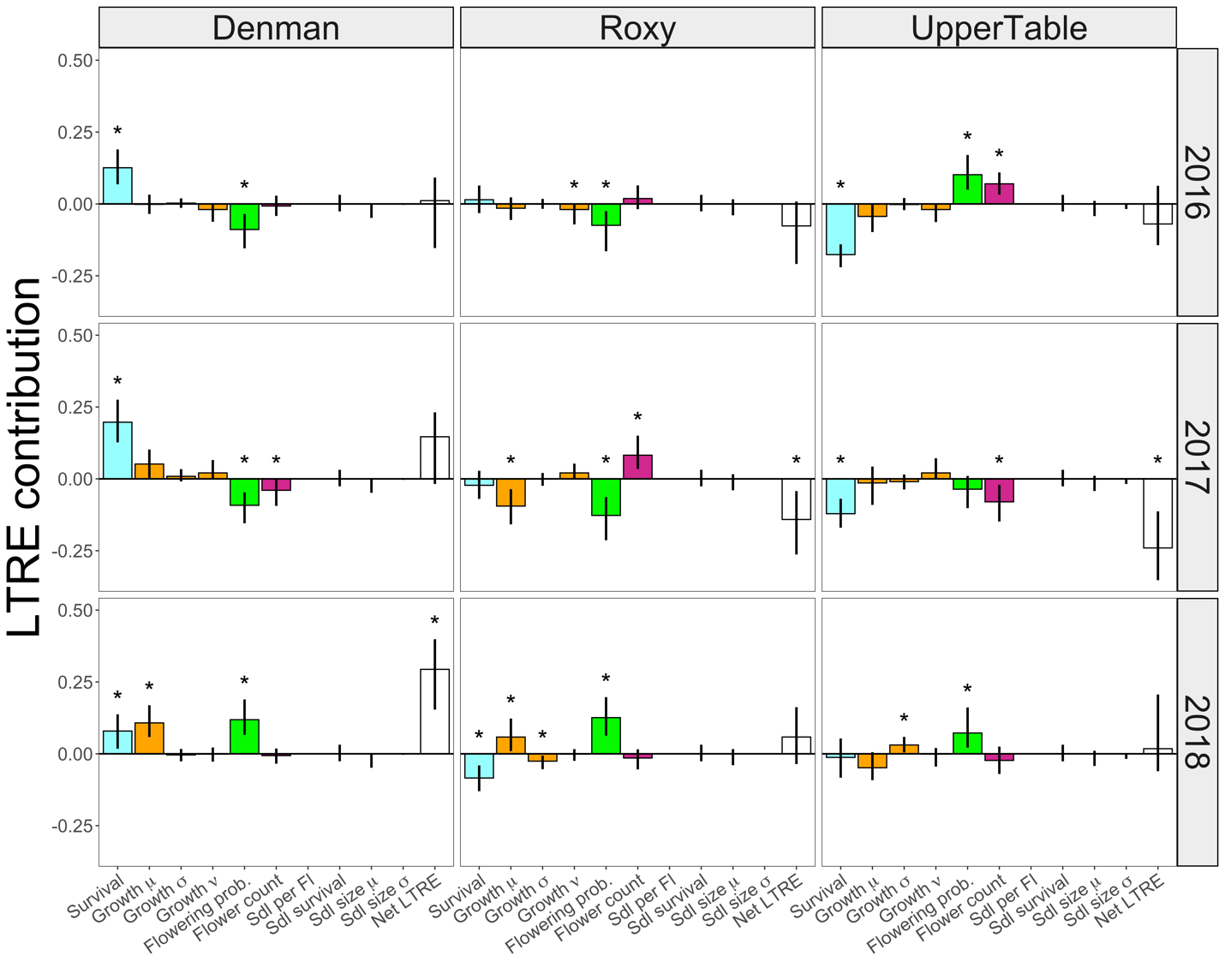


**Figure S13.** Life table response experiment contributions (LTRE) (and 95% CI) of vital rates across sites and years. LTRE values describe the contribution of different vital rates to variation in *λ* across site-years; a positive contribution means that vital rate had a positive influence on *λ* in that site or year relative to the mean integral projection model. Asterisks denote when 95% confidence intervals do not overlap 0, indicating a significant contribution. LTRE contributions for each vital rate model were summed across the intercept and slope. *μ* = mean size estimate, *σ* = size variance, *ν* = growth skewness.

**LITERATURE CITED**

Kahle, D., and H. Wickham. 2013. ggmap: Spatial visualization with ggplot2. *R Journal* 5: 144-161.
