# Supplementary material for: Spatiotemporal variation in population dynamics of a narrow endemic, Ranunculus austro‐oreganus
Source: Am J Bot. 2024 Dec 17;112(1):e16446. doi: 10.1002/ajb2.16446 (PMC11744433; doi:10.1002/ajb2.16446)
Supplement: Supplementary file 2 — Appendix S2. Supplemental Tables S1–S8. [file AJB2-112-e16446-s001.docx]

Appendix S2. Supplemental tables.

**Table S1.** Site and sample information for the three survey sites
of *R. austro-oreganus*. Data were initially reported in Hendricks
(2016).

| Site characteristic | Denman | Roxy | UpperTable |
| --- | --- | --- | --- |
| Latitude | 42.461 | 42.348 | 42.469 |
| Elevation (m a.s.l.) | 378 | 949 | 418 |
| Annual precipitation (mm) | 564.2 | 781.7 | 575.2 |
| *R. occidentalis* present | Yes | No | Yes |
| Soil depth (cm) | 29 | >80 | 17 |
| Soil texture: sand (%) | 18.0 | 7.5 | 33.7 |
| Soil texture: clay (%) | 43.4 | 56.0 | 19.2 |
| Soil texture: silt (%) | 38.5 | 36.5 | 47.1 |
| Total soil nitrogen (%) | 0.11 | 0.09 | 0.16 |
| Total soil carbon (%) | 1.24 | 1.19 | 2.20 |
| No. of unique plants surveyed | 281 | 257 | 366 |

**Table S2.** Model coefficients and standard errors (parentheses) for the best-fit vital rate models for *Ranunculus austro-oreganus*. Coefficients and errors are presented in the link scale (i.e., they are untransformed). The model’s error structure is specified as m*odel type*. For categorical predictors, the factor is specified in brackets; the reference level for categorical predictors is: Site = Denman, Year = 2016. Reported levels for “Year” is the second year of each vital rate transition. The seedling and adult growth models are generalized additive models with multiple responses modeled: µ is the mean response, σ is error, and ν is the skewness parameter. Blank cells are predictors included in the maximal model that were not in the best model, and grayed-out cells are predictors not tested for each specific vital rate.

| Model name | Seedling survival | Seedling growth *µ* | Seedling growth σ | Survival | Growth *µ* | Growth *σ* | Growth *ν* | Flowering probability | Flower number | Seedlings per flower |
| --- | --- | --- | --- | --- | --- | --- | --- | --- | --- | --- |
| Model type | Binomial | Normal | - | Binomial | Skewed normal | - | - | Binomial | Negative binomial | Normal |
| Link function | logit | identity | log | logit | identity | log | identity | logit | log | identity |
| Intercept | 0.32 (0.16) | 4.29 (0.08) | -0.24 (0.07) | -1.76 (0.65) | 3.99 (0.35) | 0.27 (0.41) | 0.36 (1.25) | -13.99 (1.64) | -0.55 (0.32) | 0.64 (0.18) |
| Size |  |  |  | 0.87 (0.13) | 0.29 (0.07) | -0.14 (0.08) | -0.46 (0.27) | 2.53 (0.31) | 0.33 (0.06) |  |
| Site (Roxy) |  |  |  | -0.83 (0.31) | -0.31 (0.32) | 0.33 (0.36) |  | -5.01 (1.60) | 0.12 (0.13) |  |
| Site (UpperTable) |  |  |  | -2.26 (0.28) | -1.27 (0.33) | -1.49 (0.44) |  | 1.05 (1.43) | 0.36 (0.11) |  |
| Year (2017) |  |  |  | 3.33 (1.18) | 1.60 (0.44) | 0.33 (0.50) | -8.87 (2.39) | 12.21 (1.77) | -0.15 (0.14) |  |
| Year (2018) |  |  |  | 0.53 (1.22) | 1.15 (0.46) | -0.05 (0.48) | -8.20 (2.24) | 12.89 (1.68) | 0.01 (0.10) |  |
| Size × Site (Roxy) |  |  |  |  | 0.06 (0.06) | -0.06 (0.07) |  | 1.02 (0.30) |  |  |
| Size × Site (UpperTable) |  |  |  |  | 0.24 (0.07) | 0.33 (0.09) |  | 0.09 (0.28) |  |  |
| Size × Year (2017) |  |  |  | -0.61 (0.23) | -0.30 (0.09) | -0.08 (0.10) | 1.94 (0.46) | -2.44 (0.34) |  |  |
| Size × Year (2018) |  |  |  | -0.19 (0.24) | -0.17 (0.09) | 0.02 (0.09) | 1.69 (0.43) | -2.24 (0.32) |  |  |
| Site (Roxy) × Year (2017) |  |  |  | -0.81 (0.47) | -0.40 (0.08) | 0.05 (0.11) |  | -0.39 (0.41) | 0.45 (0.21) |  |
| Site (UpperTable) × Year (2017) |  |  |  | -0.12 (0.46) | -0.07 (0.11) | 0.12 (0.14) |  | -1.07 (0.38) | -0.55 (0.21) |  |
| Site (Roxy) × Year (2018) |  |  |  | -0.39 (0.43) | -0.11 (0.10) | 0.16 (0.12) |  | -0.06 (0.38) | -0.16 (0.16) |  |
| Site (UpperTable) × Year (2018) |  |  |  | 1.58 (0.47) | -0.34 (0.10) | -0.35 (0.14) |  | -1.88 (0.38) | -0.44 (0.17) |  |
| Dispersion parameter |  |  |  |  |  |  |  |  | 7.49 (1.47) |  |

**Table S3.** Candidate models for adult survival. We included all models with within ΔAICc ≤ 2 of the minimum and with cumulative AICc weights < 0.95. “X” indicates which categorical predictors are included in each model, and + or – shows when a continuous predictor (size) has a positive or negative slope, respectively. The best-fit model is bolded.

| Survival | | | | | | | | | | | | | |
| --- | --- | --- | --- | --- | --- | --- | --- | --- | --- | --- | --- | --- | --- |
| Intercept | Size | Site | Year | Size × Site | Size × Year | Site × Year | Size × Site × Year | df | logLik | AICc | ΔAICc | Weight | Cum. weight |
| **X** | **+** | **X** | **X** |  | **X** | **X** |  | **12** | **-706.84** | **1437.88** | **0** | **0.569** | **0.569** |
| X | + | X | X | X | X | X |  | 14 | -705.63 | 1439.52 | 1.64 | 0.251 | 0.820 |
| X | + | X | X |  |  | X |  | 10 | -710.39 | 1440.91 | 3.03 | 0.125 | 0.945 |

**Table S4.** Candidate models for adult flowering probability. We included all models with within ΔAICc ≤2 of the minimum and with cumulative AICc weights < 0.95. The best-fit model is bolded. Because we defined the best model as the model with ΔAICc ≤2 with the fewest terms, the best model is not the model with the highest weight. In this case, the best model added to a cumulative weight >0.95; we still included this model in the candidate model list, as its weight was considerable, at 0.321. X indicates which categorical predictors are included in each model, and + or – shows when a continuous predictor (size) has a positive or negative slope, respectively.

| Flowering probability | | | | | | | | | | | | | |
| --- | --- | --- | --- | --- | --- | --- | --- | --- | --- | --- | --- | --- | --- |
| Intercept | Size | Site | Year | Size × Site | Size × Year | Site × Year | Size × Site × Year | df | logLik | AICc | ΔAICc | Weight | Cum. weight |
| X | + | X | X | X | X | X | X | 18 | -805.51 | 1647.44 | 0 | 0.677 | 0.677 |
| **X** | **+** | **X** | **X** | **X** | **X** | **X** |  | **14** | **-810.33** | **1648.93** | **1.49** | **0.321** | **0.998** |

**Table S5.** Candidate models for flowering number. We included all models within ΔAICc ≤2 of the minimum and with cumulative AICc weights <0.95. The best-fit model is bolded. X indicates which categorical predictors are included in each model, and + or – shows when a continuous predictor (size) has a positive or negative slope, respectively.

| Flower number | | | | | | | | | | | | | |
| --- | --- | --- | --- | --- | --- | --- | --- | --- | --- | --- | --- | --- | --- |
| Intercept | Size | Site | Year | Size × Site | Size × Year | Site × Year | Size × Site × Year | df | logLik | AICc | ΔAICc | Weight | Cum. weight |
| **X** | **+** | **X** | **X** |  |  | **X** |  | **11** | **-1114.99** | **2252.49** | **0** | **0.424** | **0.424** |
| X | + | X | X | X |  | X |  | 13 | -1113.21 | 2253.13 | 0.63 | 0.309 | 0.733 |
| X | + | X | X | X | X | X |  | 15 | -1112.07 | 2255.07 | 2.58 | 0.117 | 0.850 |

**Table S6.** Candidate models for seedling survival. We included all models with within ΔAICc ≤2 of the minimum and with cumulative AICc weights <0.95. X indicates which categorical predictors are included in each model.

| Seedling survival | | | | | | | |
| --- | --- | --- | --- | --- | --- | --- | --- |
| Intercept | Site | df | logLik | AICc | ΔAICc | Weight | Cum. weight |
| **X** |  | **1** | **-106.83** | **215.7** | **0** | **0.856** | **0.856** |

**Table S7.** Candidate models for seedling growth. We included all models with within ΔAICc ≤ 2 of the minimum and with cumulative AICc weights < 0.95. X indicates which categorical predictors are included in each model. The letter *µ* corresponds to terms used to predict the mean size; *σ* is the standard deviation. The best-fit model is bolded. Because we defined the best model as the model with ΔAICc ≤2 with the fewest terms, the best model is not the model with the highest weight.

| Seedling growth | | | | | | | | | |
| --- | --- | --- | --- | --- | --- | --- | --- | --- | --- |
| Intercept  *µ* | Intercept *σ* | Site *µ* | Site *σ* | df | logLik | AICc | ΔAICc | Weight | Cum. weight |
| X | X | X | X | 6 | -102.25 | 217.50 | 0 | 0.515 | 0.515 |
| **X** | **X** |  |  | **2** | **-107.35** | **218.85** | **1.34** | **0.263** | **0.778** |
| X | X | X |  | 4 | -106.05 | 220.57 | 3.06 | 0.111 | 0.889 |

**Table S8.** Candidate models for seedlings per flower. We included all models within ΔAICc ≤2 of the minimum and with cumulative AICc weights <0.95. X indicates which categorical predictors are included in each model.

| Seedlings per flower | | | | | | | | |
| --- | --- | --- | --- | --- | --- | --- | --- | --- |
| Intercept | Site | Year | df | logLik | AICc | ΔAICc | Weight | Cum. weight |
| **X** |  |  | **2** | **-6.45** | **18.90** | **0** | **0.981** | **0.981** |

**LITERATURE CITED**

Hendricks, L. B. 2016. The performance of four native perennial forb species along a climate gradient in Pacific Northwest Prairies.MS thesis, University of Oregon, Eugene, Oregon, USA.
