# Supplementary material for: Spatiotemporal variation in population dynamics of a narrow endemic, Ranunculus austro‐oreganus
Source: Am J Bot. 2024 Dec 17;112(1):e16446. doi: 10.1002/ajb2.16446 (PMC11744433; doi:10.1002/ajb2.16446)
Supplement: Supplementary file 3 — Appendix S3. Supplemental Table S9. [file AJB2-112-e16446-s002.pdf]

Table S9. Candidate models for seedling growth. We included all models with within ΔAICc ≤ 2 of the minimum or with cumulative AICc weights < 0.95. X indicates which categorical predictors are included in each model, and + or – shows when a continuous predictor (size) has a positive or negative slope, respectively. The best-fit model is bolded. The μ symbol corresponds to terms used to predict the mean size, while σ is the standard deviation, and ν is the skew parameter.

Intercept μ	Size μ	Site μ	Year μ	Size * Site μ	Size * Year μ	Site * Year μ	Size * Site * Year μ	Intercept σ	Size σ	Site σ	Year σ	Size * Site σ	Size * Year σ	Site * Year σ	Size * Site * Year σ	Intercept ν	Size ν	Site ν	Year ν	Size * Site ν	Size * Year ν	Site * Year ν	Size * Site * Year ν	df	logLik	AICc	ΔAICc	weight	Cumulative weight
**X**	**+**	**X**	**X**	**X**	**X**	**X**		**X**	**-**	**X**	**X**	**X**	**X**	**X**		**X**	**-**		**X**		**X**			**34**	**-943.17**	**1956.30**	**0.00**	**0.533**	**0.506**
X	+	X	X	X	X	X		X	-	X	X	X	X	X	X	X	-		X		X			38	-940.64	1959.72	3.43	0.096	0.598
X	+	X	X	X		X		X	-	X	X	X	X	X		X	+		X		X			32	-947.81	1961.34	5.04	0.043	0.638
X	+	X	X	X		X		X	-	X	X	X		X		X	-		X		X			30	-950.10	1961.71	5.42	0.036	0.672
X	+	X	X	X	X	X		X	-	X	X	X	X	X	X	X	-	X	X		X			40	-939.60	1961.90	5.61	0.032	0.703
X	+	X	X	X		X		X	-	X	X	X		X		X	-	X	X		X			32	-948.36	1962.45	6.15	0.025	0.726
X	+		X		X			X	-	X	X	X	X			X	-	X	X		X	X		28	-952.98	1963.28	6.99	0.016	0.741
X	+	X	X	X	X	X		X	-	X	X	X	X	X		X	+		X					32	-948.86	1963.46	7.16	0.015	0.755
X	+	X	X	X		X		X	-	X	X	X	X	X	X	X	+							32	-948.98	1963.68	7.38	0.013	0.768
X	+		X		X			X	-	X	X	X	X	X		X	-	X	X		X	X		32	-949.01	1963.74	7.44	0.013	0.780
X	+	X	X	X	X	X		X	-	X	X	X	X			X	-		X		X			30	-951.15	1963.83	7.53	0.012	0.792
X	+		X		X			X	-	X	X	X	X	X		X	-	X	X	X	X	X		34	-947.12	1964.19	7.90	0.010	0.802
X	+							X	-	X	X	X	X	X		X	+	X	X	X		X		28	-953.61	1964.54	8.24	0.009	0.810
X	+	X	X	X		X		X	-	X	X	X	X	X	X	X	+	X	X		X			38	-943.17	1964.79	8.49	0.008	0.817
X	+	X	X		X	X		X	-	X	X	X	X	X	X	X	-	X		X				36	-945.50	1965.19	8.90	0.006	0.823
X	+	X	X	X	X	X		X	-	X	X	X	X	X	X	X	-	X	X	X	X			42	-939.16	1965.31	9.01	0.006	0.829
X	+	X	X		X	X		X	-	X	X	X		X		X	-	X	X		X			32	-949.83	1965.40	9.10	0.006	0.834
X	+	X	X			X		X	-	X	X	X	X	X		X	+							26	-956.22	1965.58	9.28	0.005	0.839
X	+	X	X	X	X	X		X	-	X	X	X	X	X		X	-	X	X	X				36	-945.77	1965.72	9.43	0.005	0.844
X	+	X	X	X	X	X	X	X	-	X	X	X		X		X	-	X	X		X			40	-941.52	1965.73	9.44	0.005	0.848
X	+							X	-	X	X		X	X		X	+	X	X	X		X		26	-956.43	1966.00	9.70	0.004	0.852
X	+	X	X		X	X		X	-	X	X	X	X	X	X	X	-	X	X	X				38	-943.80	1966.04	9.75	0.004	0.856
X	+	X	X		X	X		X	-	X	X	X	X	X		X								27	-955.41	1966.05	9.75	0.004	0.860
X	+	X	X		X	X		X	-	X	X	X	X	X		X								27	-955.41	1966.05	9.75	0.004	0.864
X	+	X	X		X	X		X	-	X	X	X	X	X		X								27	-955.41	1966.05	9.75	0.004	0.868
X	+							X	-	X	X	X	X	X		X	-	X	X	X	X	X		30	-952.36	1966.23	9.93	0.004	0.871
X	+	X	X	X	X			X	-	X	X	X	X	X		X	-	X	X		X	X		36	-946.12	1966.42	10.12	0.003	0.874
X	+	X	X	X	X	X		X	-	X	X	X	X	X	X	X	+		X					36	-946.14	1966.47	10.18	0.003	0.877
X	+		X		X			X	-	X	X	X	X			X	-	X	X	X	X	X		30	-952.51	1966.55	10.25	0.003	0.880
X	+		X					X	-	X	X	X	X	X		X	+	X	X	X		X		30	-952.54	1966.60	10.30	0.003	0.883
X	+	X	X	X	X	X		X	-	X	X	X	X	X	X	X	+							34	-948.34	1966.62	10.33	0.003	0.886
X	+		X		X			X	+	X	X	X	X	X	X	X	-	X	X		X	X		36	-946.23	1966.65	10.36	0.003	0.889
X	+	X	X	X	X	X		X	-	X	X	X	X	X		X	+	X	X			X		38	-944.14	1966.71	10.42	0.003	0.892
X	+		X		X			X	-	X	X	X	X	X		X	-	X	X			X		30	-952.62	1966.77	10.47	0.003	0.895
X	+	X	X	X	X	X		X	-	X	X	X	X	X	X	X		X						35	-947.35	1966.77	10.48	0.003	0.897
X	+		X		X			X	-	X	X		X	X		X	-	X	X	X	X	X		32	-950.53	1966.79	10.49	0.003	0.900
X	+	X	X	X		X		X	-	X	X	X	X	X	X	X	+	X	X	X				38	-944.18	1966.79	10.50	0.003	0.903
X	+	X	X			X		X	-	X	X	X	X	X		X								25	-957.90	1966.86	10.57	0.003	0.905
X	+	X	X			X		X	-	X	X	X	X	X		X								25	-957.90	1966.86	10.57	0.003	0.908
X	+	X	X			X		X	-	X	X	X	X	X		X								25	-957.90	1966.86	10.57	0.003	0.910
X	+		X		X			X	-	X	X	X	X	X		X	-	X	X	X		X		32	-950.66	1967.05	10.76	0.002	0.913
X	+	X	X	X	X	X		X	-	X	X	X	X	X	X	X	-	X	X		X	X		44	-937.92	1967.11	10.81	0.002	0.915
X	+							X	-	X	X	X	X	X		X		X	X			X		25	-958.08	1967.22	10.93	0.002	0.917
X	+	X	X	X	X	X		X	-	X	X	X	X	X	X	X	+	X	X					38	-944.40	1967.25	10.95	0.002	0.919
X	+		X		X			X	-	X	X	X	X	X	X	X	-	X	X	X	X	X		38	-944.47	1967.39	11.09	0.002	0.921
X	+	X	X	X	X	X		X	-	X	X	X	X	X		X			X					31	-951.92	1967.46	11.16	0.002	0.923
X	+	X	X		X			X	-	X	X	X	X	X		X	-	X	X		X	X		34	-948.77	1967.49	11.19	0.002	0.925
X	+	X	X			X		X	-	X	X	X	X	X	X	X	+	X	X	X				36	-946.67	1967.52	11.22	0.002	0.927
X	+	X	X		X	X		X	-	X	X	X	X	X		X	+							28	-955.10	1967.53	11.23	0.002	0.929
X	+							X	-	X	X	X	X	X	X	X	+	X	X	X		X		32	-950.94	1967.60	11.30	0.002	0.930
X	+	X	X	X		X		X	-	X	X	X	X	X	X	X	+	X	X		X	X		42	-940.32	1967.62	11.32	0.002	0.932
X	+	X	X		X	X		X	-	X	X	X	X	X	X	X	+	X	X	X	X			40	-942.48	1967.65	11.36	0.002	0.934
X	+	X	X			X		X	-	X	X	X	X	X	X	X	-	X	X	X	X			38	-944.61	1967.67	11.37	0.002	0.936
X	+	X	X	X		X		X	-	X	X	X	X	X	X	X	+	X	X	X	X			40	-942.65	1968.01	11.71	0.002	0.937
X	+	X	X					X	-	X	X	X	X	X		X	+	X	X	X		X		32	-951.18	1968.09	11.79	0.001	0.938
X	+							X	-	X	X		X	X		X	-	X	X	X	X	X		28	-955.39	1968.10	11.81	0.001	0.940
X	+	X	X	X		X		X	-	X	X	X	X	X		X	+	X	X		X	X		38	-944.89	1968.21	11.92	0.001	0.941
X	+	X	X		X	X		X	+	X	X	X	X	X	X	X	-	X	X		X			38	-944.90	1968.23	11.93	0.001	0.942
X	+							X	-	X	X	X	X	X		X	-	X	X		X	X		28	-955.54	1968.40	12.10	0.001	0.944
X	+	X	X		X	X		X	+	X	X	X	X	X	X	X	-	X	X					36	-947.11	1968.41	12.11	0.001	0.945
X	+	X		X				X	-	X	X	X	X	X		X	+	X	X	X		X		32	-951.37	1968.47	12.18	0.001	0.946
X	+	X	X	X	X	X		X	-	X	X	X	X	X	X	X	-	X						36	-947.17	1968.54	12.24	0.001	0.947
X	+	X	X	X	X	X		X	-	X	X	X	X	X	X	X		X	X					37	-946.12	1968.56	12.26	0.001	0.948
X	+	X	X			X		X	-	X	X	X	X	X	X	X	+							30	-953.52	1968.56	12.27	0.001	0.949
